# Spirit of the Foreign Periodicals, &c.

**Published:** 1837-07-01

**Authors:** 


### Spirit of the Foreign Periodicals, &c.

### Biographical Sketch of the late Baron Dupuytren.

The life of such a man as Dupuytren ought to be well known to every young candidate for professional fame, as affording an admirable example of what energy, perseverance, and ability will do. He was born of very humble parents, in the year 1772.

At the early age of twelve years, he was induced by an officer of a regiment which was quartered in his native village, to go to Paris. Here he became acquainted with the late M. Alard; and the two young fellow-labourers commenced and prosecuted their studies with the most devoted and unwearied zeal. They had but one paltry room between them; and this served for a sleeping, as well as an eating apartment. All the furniture they had, was a table, a couple of chairs, and one bed; and their cupboard was often but very scantily provided, even with the necessaries of life. Dupuytren used to take great pleasure in his after-life, when in the zenith of his fame and success, to talk of the "res angusta domi," and various hardships which he had to submit to at the commencement of his career. He used to mention an anecdote of his being visited by the visionary enthusiast, Saint Simon, who had early perceived the vigorous talents of the medical student, and was anxious to make him a convert to his doctrines. After a long but ineffectual conversation, the *Saint* rose to take his leave; and, while Dupuytren's attention was withdrawn to another part, he took the opportunity of putting down a purse, containing 200 francs, in a corner of the apartment.

The proud spirit of the youth refused all such assistance, although he afterwards confessed that, at this time, his pecuniary means were scarcely sufficient to procure him the ordinary comforts of life.

It was in 1794, when he was only 17 years of age, that he first had an opportunity of giving proofs of ability, and an earnest of his future fame. At a public concours, he was appointed one of the "prosecteurs," or anatomical demonstrators at the Ecole de Medicine, then recently established by government. During the next five years he laboured most assiduously in the dissecting-room, and thus laid the foundation of that intimate knowledge of minute anatomy, which, perhaps, more than any other acquirement, served to render him, like Baillie, one of the greatest masters of diagnosis in disease. In 1801, he publicly contested with Dumeril for the place of "chef des travaux anatomiques," but lost it by only one vote. Six months afterwards, however, he was elected in the place of Dumeril, who had been raised to a professorship. He now commenced to lecture upon anatomy, physiology, and pathology. In 1802, Bichat, still in the flower of his age, died; and thus the field was left almost undisputed to Dupuytren, who had been educated in the same school, and had followed out the same method of investigation, as his immortal predecessor had done. In 1803, he was elected "chirurgien de seconde classe," to the Hôtel Dieu, the largest hospital in Paris. His rivals on this occasion were Roux, Tartra, and Hedeloffer. In 1808, he was made "chirurgien en chef adjoint," and three years afterwards Sabatier, the senior surgeon, having died, Dupuytren was appointed the colleague of Pelletan. At the same time he succeeded to the chair of medecine operatoire, vacant by the death of Sabatier, after a vigorous contest with M. M. Roux, Tartra, and Marjolin. The concours upon this occasion was one of the most animated and protracted, which has been ever known in Paris. For forty days, "les concurrens furent tenus en haleine;" they were required to give in written answers—in Latin as well as in French—to a multitude of questions proposed, to deliver extempore lectures on surgical and anatomical subjects, to perform operations on the dead body in presence of the examiners, and lastly, to write a thesis on a theme which had been given them.

Dupuytren had very nearly lost the election, in consequence of some delay in completing his thesis! He was generally a very slow composer, and at all times much fitter for the active practical duties of his profession, than for the meditative occupation of the study.

He was now a prominent man in the eyes of the public; surgeon of the Hôtel Dieu, and professor of operative surgery at the Ecole de Medicine. Soon after his appointment as one of the chief surgeons to the Hôtel Dieu, Pelletan withdrew, in consequence, it was said, of frequent quarrels with his junior colleague. This is not unlikely, as Dupuytren was naturally haughty and ambitious; and all who know him, were well aware that he never could endure a rival near his throne. While yet a boy, he was fond of repeating Cæsar's saying, "that that he would rather be the first man in his own village, than the second in Rome;" and throughout his after-life, the Napoleon of surgery (as Bouillaud calls him) seems to have always acted on this rather unamiable maxim. The energy, the unceasing assiduity, the devotedness of zeal, and the eminent ability with which he performed the duties of chief surgeon of the Hôtel Dieu are above all praise. He spent between four and five hours there daily, visiting and minutely examining his patients, performing operations, superintending dissections, and lecturing. His first visit was usually paid about seven o'clock in the morning, and he seldom failed to return in the evening, to watch the progress of the more interesting cases. Most faithful reports of all these were drawn up by the "internes," and afterwards corrected and arranged by himself. There are at the present time upwards of 700 thick folio volumes of these reports, the cases being all arranged in alphabetical tables, preserved at the Hôtel Dieu!

In making his rounds through the hospital, he was generally reserved and taciturn; spoke little, and that little not in the most urbane manner, to the students. In all his actions, and upon all occasions he was the same calm, imperturbable being; ready and prepared for every emergency, never taken by surprise, or moved by any unforeseen danger. "Cette paix, ce calme de l'esprit, ce premier element de tout bonheur, cette divinité de l'epicurisme, Dupuytren le cherchait en tout. Aussi, dans ses leçons, dans ses operations, rien n'etait remis à la fortune; tout etait muri, calculé, approfondi, prevu."

If there were any unusual symptoms in a case, if the diagnosis was obscure and uncertain, he refrained from giving any opinion at first; he hesitated for some days, thinking and reasoning upon it; and when at length he had arrived at some satisfactory conclusions, his clinical lecture on the subject was a model of excellence, full of impressive thought, and stored with the soundest practical wisdom.

Dupuytren was unquestionably one of the most *medical* of all surgeons. It was not the *art* alone, it was the *science* of surgery, which he explored so much more deeply than his contemporaries. Having his mind enriched with the most consummate knowledge of general and descriptive anatomy, and being intimately acquainted with the whole range of physiology and pathology, he took an enlarged view of every case, tracing it back to its source, discovering the cause which had produced it, the effects which the constitution, temperament, or casual circumstances had occasioned, the changes which it had already produced, and the probable event of its progress and termination. Although one of the most accomplished operators of his time, he never sought for occasions to display his dexterity; it was a greater triumph to him to supersede the necessity of an operation, than even to have performed it well. In the use of the knife, he was always cool and calm; bold and rapid when there was no uncertainty or possible chance of mistake, as in performing amputation of the limbs, and the excision of certain tumors; slow, but steadily sure in the more delicate operations of his art.

No casualty ever disturbed his equanimity. On one occasion he had the misfortune to open an aneurismal tumor, supposing it to be an abscess.—Without hesitation and embarrassment, he at once proceeded to tie the artery higher up—the case did well. Every one has heard of the case of sudden death about sixteen years ago, during the excision of a large tumor from the neck of a young woman; the air entered the external jugular vein, and the girl, after uttering a few moans, died on the table. Dupuytren was the operator: "il est jeté dans une meditation profonde; le malheur devient pour lui le texte d'une des plus belles leçons qu'on ait jamais entendues: l'apropos l'inspirait; le sujet avait saisi les esprits; et surmontant ainsi son propre trouble pour expliquer ce tragique evenement, il rejette sur les capricieuses lois de la nature ce que la malignité eut imputé à son imprudence." Dupuytren was remarkably adroit at reducing dislocations. No one knew better how to divert the attention of the patient, and thus to take off in part the resistance of the muscles. We shall mention only one case.

A woman was brought into the hospital with dislocation of the shoulder. Several unsuccessful attempts to reduce it had been already made. Dupuytren, when the apparatus was ready, began to question the patient how she had met with the accident, and as she was answering, said, "your son told me you were drunk at the time." "A ces paroles, la mere indignée tombe dans une sorte d'aneantissement, et le bras est remis."

The immense private practice, which Dupuytren enjoyed, increased the sphere of his observation.* He was the "facilé princeps" of his time. His reputation was not confined to his own country; it was European. Patients from all parts of the Continent came to Paris to consult him.

He was honoured with the confidence of two kings, elevated to the rank of a Baron, decorated with various titles of distinction, and courted and caressed by the most lofty and distinguished of his country. "Que manquait-il donc," exclaims his eulogist, "à son bonheur. Mais le bonheur n'est point dans la situation; il est dans le caractere; et Dupuytren n'etait pas né pour etre heureux. Tout ce qu'il avait souhaité, il l'avait à profusion; et n'y sentait que vide et desespoir."* In 1833, his health suffered a severe shock; and in the following Spring he was induced to travel into Italy. At Rome he met with M. Esquirol, who, observing the restlessness and impatience of his colleague to return to Paris, asked of him the reason. "Qui vous presse?—Je songe à Hôtel Dieu, repond Dupuytren. Vous l'avez laissé dans d'habiles mains, reprend Esquirol. Oui, replique Dupuytren, mais mon devoir." He soon returned to Paris; but it was only to linger out a few months of suffering. He died in the February following at the age of 57. On dissection, a considerable effusion was found in the cavity of the thorax, and the brain exhibited some traces of "foyers apoplectiques."

The fortune which Dupuytren had amassed was immense; equal perhaps to what had ever been made by professional exertions. It amounted to nearly £250,000. He bequeathed the sum of £10,000. to the establishment of a museum, and a professorship on pathological anatomy.

We have not alluded to the writings of Dupuytren. He has not done much himself as an author. With the excep-

---

* Besides his private and hospital practice, Dupuytren had ample opportunities of studying gunshot wounds, and the other casualties of military surgery, in 1814, when he was appointed surgeon to his wounded countrymen, after the battle of Montmartre. The "glorious days" of July 1830 also furnished him with a vast field for displaying his consummate surgical abilities.

* We suppose that M. Pariset alludes to the domestic infelicity of Dupuytren. His lady, it appears, left her home;—and from that hour, the haughty spirit of his temper seemed to have taken on all the austerity of a misanthropic melancholy.

tion of some memoirs on fractures of the fibula, on artificial anus, on calculus and the operation of lithotomy, and of several reports and "eloges" delivered at the Academy, we must look for the record of his doctrines and practice in the writings of his pupils and associates—in the Repertoire d'Anatomie of Breschet and Royer Collard, in Sanson and Begin's edition of Sabatier's Medecine Operatoire, in the great work of Roche and Sanson, on Medico-Chirurgical Pathology, in five volumes, and in his own Leçons Orales, published by Buet, Briere de Boismont, Marx, and Paillard.

---

### BIOGRAPHICAL SKETCH OF THE LATE BARON DESGENETTES.

Desgenettes was born in 1762, at Alençon. He studied at Montpelier, at that time by far the most celebrated school of medicine in France, and acquired there considerable reputation by a very able thesis on the anatomy of the lymphatic system. In 1793, he entered the military service of his country, and being sent to Frejus, he there, for the first time, met with "le petit cannonier, un fier militaire," the young Buonaparte. Three years afterwards, he accompanied the fleet, which carried "un autre Cæsar et sa fortune," to the shores of Egypt, as chief physician of the forces. The zeal, the courage, and professional skill, with which he ever fulfilled the duties of his arduous situation, are well known to the world. It was from him, as much as from the General himself, that the army acquired that spirit of dauntless bravery and intrepid endurance, which carried them along victorious through the sickly plains of Egypt and Syria. The soldiers had been greatly alarmed by the reports of the dreadful contagiousness of the plague. Desgenettes perceived at once the necessity of calming their fears, by one bold and decided step. For this purpose, he had recourse to an expedient, "aussi sublime que perilleux"—that of inoculating himself with the virus from a pestilential bubo, in presence of them all. He escaped the infection; and thus by a single daring, and most hazardous experiment, he not only dissipated the fears, but acquired the unbounded confidence of the whole army.

It was soon after this, that he had another opportunity of exhibiting a nobleness of character, which cannot be too often mentioned, or too warmly eulogised—we allude to his conduct on the occasion of Buonaparte's proposal to administer opium to the dying soldiers at Acre. We shall give the anecdote in Desgenettes' on words. "The General summoned me to his tent very early one morning. He was alone, with his chief aide-de-camp. After a short preamble, he said to me, 'were I in your place, I should be inclined to put an end at once to the sufferings of the plague-patients, and to the danger of their infecting the rest of the troops, by giving them opium.' I answered simply, my duty is to save." (Mon devoir, a moi, c'est de conserver.)

Upon this, the General proceeded with the greatest calmness to explain the motives of his suggestion, saying, that what he advised for others, he should certainly wish might be done to himself, were he similarly placed. He alluded, also, to the probable fate of his unhappy soldiers, if they were left to fall into the hands of the merciless Turks.

In conclusion he said, 'I do not wish to overcome your scruples, but I shall probably find other persons, who will better appreciate my intentions.'

General Berthier was present during this conversation; he remained silent; but afterwards he cordially approved of my conduct on the occasion.

It was not, until after our return from Jaffa, that the opium was administered to between 25 and 30 of the sick soldiers. Some of them rejected it by vomiting, and ultimately recovered."

Desgenettes remained in Egypt with the army, after Buonaparte had made his escape to France. In 1802, he returned to Paris, was cordially received by the First Consul, and appointed by him Chief Physician of the Military

Hospital, and Inspector General of the Army. About the same time he published his "Histoire Medicale de l'Armée de l'Orient,"—a model of historical and professional description.

In 1805 he visited Spain, for the purpose of examining the epidemic fever, which raged at Cadiz and Malaga; and in the following year, he accompanied the French army in their memorable campaigns in Prussia, Poland, &c.

He was present also in the unfortunate expedition to Russia, and on one occasion at Moscow, displayed to his master the same firmness and independence of spirit, which he had shewn ten years before in Egypt.

Buonaparte had ordered the Foundling Hospital of Moscow to be converted into barracks for the troops.—Desgenettes remarked to him that he feared that posterity might compare him to Herod. "Herod," exclaimed the Emperor, "how Herod! What has he to do here, and in what can I be like him." "In the massacre of the innocents," replied Desgenettes.—The answer was a happy one, for the order was immediately countermanded.

In the disastrous retreat from Russia, Desgenettes was taken prisoner; but he was liberated soon after by the special orders of the Emperor Alexander. He was present at the battle of Leipsic. The Bourbon Government seems to have been unfriendly towards him, as he was deprived of several of his posts upon their first restoration.

During the "Hundred Days," Desgenettes was appointed by Napoleon chief physician of the army and of the Imperial Guard; and accompanied his master to the fatal field of Waterloo.

He was again in disgrace, when Louis was a second time raised to the throne of France; but in 1819, he was recalled, and made one of the Council of Health for the army.

During, however, the preceding four years he had been most zealously engaged in fulfilling the duties of Professor of Hygiene of Medical Physics to the Faculty of Medicine in Paris. His vast erudition, his zeal and energy, and eloquence of his language, attracted universal admiration.

In 1822, he along with ten other professors,* was deprived of their chairs by a most iniquitous act of the government, when the ordonnance for the suppression of the Faculty of Medicine was issued.

In alluding to this event in the history of Desgenettes, his eulogist, M. Bouillaud, cannot refrain from breaking forth in most oratorical indignation. "Illustrious victims, bear up against this cruel trial. He who presides over the destinies of the world will not permit that the sacred laws of justice should be long violated with impunity, and he will teach, in due season, the kings of the earth themselves, many a sore and bitter lesson."

This allusion points to the "glorious revolution of July," when Desgenettes and the other ejected professors were restored to their dignities. He continued for several years to perform the duties of his chair, although now upwards of seventy years of age. During lecture one day, he was struck with a slight attack of apoplexy, "comme un general est frappé d'un coup de feu sur le champ de bataille;" and although he recovered after a few weeks, he never resumed his professorial labours.

He occupied his spare time in writing memoirs of his interesting and most eventful life, of which the third volume has recently appeared. His health, although infirm, continued to be tolerably good until the month of last February, when he most suddenly had another attack of apoplexy, from which he never recovered. "Mais il est temps de vous saluer d'un dernier adieu, venerable collégue. Adieu donc, trois fois adieu, O Desgenettes! Puissent vos precieux restes, se ranimant un instant, n'etre pas tout-a-fait insensible aux accens d'une voix respectueuse et devouée."—*La Presse Medicale.*

---

* Among the ejected professors were Pinel, Chaussier, and Jussien. The conduct of the French Government appears to have been most arbitrary and impolitic.

INFLAMMATION OF THE SPINAL MARROW.—ALLEGED FREQUENCY OF THIS DISEASE IN AGUES.—GENERAL REMARKS ON ITS MORE PROMINENT SYMPTOMS.

A middle-aged woman, of a robust and plethoric habit, had been exposed to wet and cold, while in a state of free perspiration. Two or three days afterwards she was seized with shiverings, and the usual signs of pyrexia: to these were added pricking and shooting pains in the lower limbs, with occasional convulsive twitches and violent dartings, which the patient compared to smart electrical shocks. When these symptoms abated, the limbs were benumbed, and almost quite powerless. Pain, accompanied with a sense of great heat, was felt along the whole tract of the spinal column.

The breathing became, in the course of a few days, difficult and uneasy; and the upper limbs were affected, although to a less degree, in the same manner as the lower ones.

The physician, who first visited the patient, had viewed her case as one of rheumatism, and the practice, which he had followed, had not been attended with success. She was taken to St. Peter's Hospital at Brussels, and put under the care of Dr. Mons, the chief physician. All the symptoms above described were present at the date of her admission; the pulse was small, rapid, and sometimes irregular; and the breathing had become much more oppressed than hitherto. The slightest pressure over the dorsal and lumbar vertebræ occasioned severe pain and an aggravation of all the symptoms. A large quantity of blood was taken by cupping over the most tender parts of the spine, and the bowels were briskly purged. This treatment required to be repeated several times, before the cure was completed.

*Case* 2. A young man had, three weeks before his admission into the hospital, been suddenly seized with a violent fit of shivering, which had supervened without any apparent cause. Soon afterwards he experienced severe pain in the back, cramps and a sense of pricking and stretching in the lower limbs, which were at the same time benumbed and so useless, that he could not stand unsupported.

These symptoms ceased altogether in the course of an hour or so; but the patient was left in a state of great depression and weakness. Two similar attacks returned within the course of the following fortnight. The present—the fourth—attack has been the most severe of all, and was at first accompanied with symptoms of gastritis. This attack did not cease spontaneously.—[It is observed in the report, "la maladie intermittente etait devenue quotidienne."] The spine from the eighth dorsal to the lower lumbar vertebræ was found to be very painful, when pressed upon. Ten cupping instruments were applied, and a large quantity of blood was thus withdrawn. A few doses of purgative medicine completed the cure.

A third case of Myelitis is given. The symptoms were not so well marked as in the preceding two instances. A youth had for about six weeks been suffering from flying pains in his legs, especially about the knees, and in the back. He could not walk, nor even stand alone, without support. The toes and fingers were affected with spasmodic twitchings. The general health was much deranged, as appeared from the utter loss of appetite, feverishness, slight diarrhœa, &c. On examining the back, pressure over the lumbar vertebræ was found to give great pain, and to aggravate all the unpleasant feelings in the limbs. By applying cupping-glasses to the back, and leeches to the anus, along with the use of aperient and diaphoretic medicines, the boy was quickly relieved of all the unpleasant symptoms.

In the following two cases, the inflammation of the spinal marrow was coexistent with intermittent fever. It appears that the Belgium physicians have of late observed this complication very frequently, and some of their writers have suggested that many of the most obstinate cases of ague are very generally associated with, if not more immediately dependent upon, an inflammatory affection of the medulla spinalis; and that they are more quickly and effectually relieved by local remedies applied to the spine than by any internal medication.

The first case, which we find reported, was one of no great severity, and does not therefore require to be given in detail. The second one was much more severe. A man, 55 years of age, had during a period of eight months experienced repeated attacks of tertian ague. When admitted into the hospital he had been suffering upwards of three weeks from regularly recurring paroxysms, in spite of the free use of quinine, and of other remedies. There were present symptoms of slight gastro-enteritis, attended with occasional attacks of pain in the abdomen, so acute that they forced the patient to bend himself double during the paroxysm. These attacks usually subsided on the expulsion of a quantity of wind from the bowels. From the abdomen the pain appeared to extend down along the lower limbs, following the line of the sciatic nerves, and causing convulsive twitches, and a sense of pricking and dragging in both legs.

On passing the finger along the spine, that portion of it from the tenth dorsal to the fourth lumbar vertebræwas found to be very tender.

One large cupping, and the use of appropriate remedies for the relief of the gastric irritation, quite dissipated all these unpleasant symptoms, and appeared to cure the ague at the same time.

The preceding cases are drawn from the Clinique of Dr. Mons at the Brussels Hospital. In the same number (for last October) of the Bulletin Medical Belge, there is a communication from Dr. Steyls of Lacken, on the continued and intermittent fevers of that place. He alludes in a very pointed manner to the great frequency of a fixed pain at some portion of the spine, generally over the lower dorsal and upper lumbar vertebræ, in the severe and protracted cases. Very frequently, perhaps generally, this symptom is of purely nervous origin and ceases spontaneously; but in some instances it becomes chronic, and then is apt to be associated with unpleasant abdominal disturbance, with dyspnœa and cough, with intense headache, and occasionally with neuralgia of the sciatic and other nerves. Under these circumstances, an intermittent fever is very apt to become of a remittent or even of a continued form, the type being at the same time alarmingly adynamic.

In such a case we may have reason to suspect that a subacute inflammation of some portion of the spinal cord is present, and that this complication is in truth the cause of the extreme obstinacy of the irregular pyrexia. We are not aware that the coexistence of Myelitis with certain forms of intermittent fever has been particularly noticed by any author; and, as this species of inflammation has probably not been much studied by the bulk of medical men, in consequence of the obscurity of its symptoms, it may be useful to describe briefly its more prominent character.

Myelitis is much more frequently of a chronic than of an active or acute form. The symptoms of the latter are usually so distinct and well-marked, as to lead the physician at once to a correct diagnosis of the case. There is pain, sometimes sharp, but more frequently of a dull oppressive character—except on sudden motion or on firm compression of the part—at one part of the spine. From this point, it extends upwards and downwards, and is accompanied with general malaise, and with a very marked diminution of all the physical energies. The pain is always aggravated by pressure with the finger, and often also by applying a sponge or towel wrung out of hot water over the part.

The brain generally sympathises very soon with all affections of the spinal marrow—hence the headache, confusion, giddiness, disturbance of vision, and other symptoms, so characteristic, it has been commonly supposed, of cerebral diseases, that the real seat of the morbid action is often overlooked. In all cases, therefore, where the usual train of head-symptoms has been present for a length of time, it is proper to have our attention directed to the state of the medulla spinalis, by a very careful examination of the vertebral column. The movements of the extremities, more especially of the lower ones, are almost always more or less disturbed, whenever the spinal marrow is inflamed.—The patient experiences either a dull numbness in them, or sharp darting pains in different parts, extending often along their whole extent. There is no outward or visible marks of any local affection in the limbs themselves; there is no redness, or heat, or swelling.—The pain is often not constant, but recurring in sharp paroxysms; and very generally it is much more severe along the course of the large nerves, and more especially at the extremities of their branches, than in other parts. Hence the ankles and the feet are more frequently complained of, than the legs, or the thighs.

When the upper extremities suffer, there is usually a co-existent affection of the respiratory functions. The patient finds his breathing uncomfortable and oppressed; there is cough, either slight or moist, or very shrill and loud, and not accompanied with expectoration. The dyspnœa often becomes suddenly aggravated in paroxyms, which last for a longer or shorter period of time. These paroxysms may have all the severity of an attack of angina pectoris—there is an insufferable pain at some part of the chest, which the patient, however, cannot point out distinctly; he feels as if he could not draw in the air, and he gasps with most ineffectual attempts.

On examining the chest by auscultation, there is often no irregularity either of the breathing, or of the heart's action discoverable. The attack, therefore, may be considered as of a purely nervous character. Even when, under such circumstances, the primary seat of the disease is not in the spinal marrow, but is altogether in the thoracic viscera, great relief may be obtained by applying counter-irritant remedies to the upper and middle part of the vertebral column. A blister to the nape of the neck, or between the shoulders, will act much more powerfully in relieving this description of dyspnœa, than if it was applied to the sternum or side of the chest.

In practice we often meet with cases of most troublesome cough, unconnected apparently with any positive disease of the chest, where the ordinary remedies altogether fail of affording relief. The cough is shrill, loud, and barking; sometimes it is so severe, that the paroxysms resemble those of genuine pertussis. Such cases are not unfrequent in young unmarried females.—By far the best remedy is a blister between the shoulders, and the internal use of some deobstruent purgative, such as the decoct. aloes composit: alone, or with an alcali. In truth, many cases of chronic dry catarrh, of chorea, hysteria, and of asthma, are, if not directly cured, greatly modified, and much aggravated by the co-existence of spinal irritation.

We have not yet alluded to the abdominal distress which usually accompanies chronic myelitis. The most common symptom is a sensation of gnawing, uneasiness, and sickening anxiety at the præcordia; accompanied sometimes with a very troublesome vomiting. It seldom amounts to a sharp or pungent pain, except when it is seated lower down; and then it is not unfrequently mistaken for genuine colic.—The bowels are usually more or less constipated; there is little or no appetite; the bowels are oppressed with flatulence, and the abdominal surface is often tense, hard, and knotty.

The secretion of the urine also is often much diminished, and the quality of the urine is, at the same time, very much altered. It is apt to be thick, loaded with mucus, has often a peculiar offensive smell, and is likely to become more quickly putrid than usual. It was Sir Benjamin Brodie, we believe, who first distinctly proved that the urine generally acquires a strong tendency to the phosphatic dyscrasis after injuries of the spine. The same phenomenon is frequently observed in cases of chronic inflammation of the marrow.

---

### Extracts from, and Remarks upon, M. Bouillaud's Clinique.

### *Typhus Fever, or Enteritis Folliculosa.*

M. Bouillaud belongs to that section of the French School, who can perceive nothing in typhus fever, but inflammation and ulceration of the gastro-intestinal mucous surface. This very partial and exclusive doctrine is not likely to be ever adopted in this country with so much indiscriminate zeal as it has been for some years past, and still is, in France. It has been very justly remarked, that the physician might with equal propriety regard the mere cutaneous eruption of the exanthemata as constituting the real essence of these fevers; as if the affection of the skin in such diseases as small-pox, malignant scarlatina, &c. was the formidable enemy, which we had to encounter, and as if any application to it could suffice to overcome the malady.

No less absurd is the doctrine of some of the continental physicians on the subject of typhus fever. It would seem from their description, that in all cases the mucous lining of the stomach or of some part of the intestines is primarily inflamed, and that the mucous glands in different parts, and more especially near the extremity of the ileum are always swollen, reddened, and ulcerated; that these lesions form, so to speak, the primary elements of the disease; that all the constitutional disturbance, such as the depression of the muscular powers, the pyrexial symptoms, the delirium, the coma, &c. &c. are merely the result of the abdominal affection; and therefore that the grand therapeutic indication is to subdue the inflammation, and to heal the ulceration of the gastro-intestinal membrane. For this purpose venæsection is to be practised; leeches in great numbers are to be applied repeatedly to the abdomen; acrid purgatives are to be avoided; nothing but soothing drinks, and emollient ptisans are to be administered; and the internal use of the chlorides is strongly recommended, with the view of checking the inflammation, and of healing the ulcerations. The use of blisters to the calves of the legs is also a favorite practice of M. Bouillaud. Such is the treatment pursued and lauded to the skies by him; and so boastful is he of his extraordinary success, that he tells us that he now looks upon typhus fever as quite as easily curable, as a mere ordinary catarrh.

Either typhus fever must be of an unusually mild character in Paris, or M. Bouillaud classifies many cases under this head, which other physicians would have excluded.

There are numerous feverish states of the system, occurring more especially in the lower orders, dependent upon exposure to cold, irregularity or deficiency of diet, to which we may add mental affliction or agitation, which will speedily recover of themselves under the soothing effects of rest, appropriate diet, and the restrictions of a well-conducted hospital. Such cases must be of very frequent occurrence in such a metropolis as Paris, where there is continually such an influx of strangers from the country and from other parts, and many of whom are exposed to the predisposing and exciting causes, mentioned above.

Our suspicions on this head are in some degree confirmed by the remark of M. Bouillaud in a former clinique, where, treating of gastro-intestinal affections, he classifics them under four heads—"embarass gastrique," "gastro-duodenite," "gastro-enterite," and "enterite folliculeuse." The reader will readily perceive that a vast number of cases may be arranged under these denominations, which have little, or no resemblance to typhus fever.

### *Intermittent Fever and Neuralgia.*

Of late years, M. Bouillaud has tried very extensively the use of full doses of Digitalis in this form of fever. He gives it inwardly; and also applies it outwardly, by sprinkling a blistered surface (usually over the spleen) with eight or ten grains of the powder. Out of six cases narrated in one of his recent reports, five recovered very quickly under this medication. One resisted its effects, and was afterwards cured by quinine.

We must confess, that we are quite at a loss to conjecture either the motive of M. Bouillaud in using digitalis for the cure of *agues*, or the mode in which he can suppose this drug can act in relieving such cases. Who, but a zealot for every thing novel and uncommon, would ever think of giving large doses of foxglove for a disease, which every one knows may be readily and safely cured with two or three scruples of quinine? But as there is no accounting for taste in other matters, so we cannot pretend to divine the motives which rule the practice of some physicians.

His treatment of *neuralgic* affections by the endermic method is much more rational. He tells us that he has succeeded in curing a number of severe cases by sprinkling the excoriated surface of the affected part with muriate of morphia. He usually commences with a fourth or a third part of a grain, and increases it to one or even to two grains. This practice he continues for a week or two.

In many cases of *palsy* too he has tried the endermic treatment with great benefit. Strychnine is the substance which he generally uses in such cases. The internal administration of this very powerful medicine, at the same time, is often of very decided advantage. No case should be given up, until a very fair trial of the strychnine, both internally and externally, has been given. M. Bouillaud assures us that he has often succeeded under the most discouraging circumstances, and mentions particularly the case of a very old man, in whom the internal use of the medicine was for some time of great benefit, but gradually seemed to lose its effect; and then the endermic method was employed with the most gratifying results.

In some very obstinate cases of *singultus* or hiccup, M. Bouillaud has had recourse to the endermic use of the muriate of morphia over the region of the stomach with very admirable effects.

Our author passes on to the consideration of affections of the *heart*, and commences with the subject of pericarditis. He reiterates his oft-repeated assertion, that almost all cases of pericarditis are of rheumatic origin, and that almost every case of severe arthritis is accompanied with a greater or less degree of this disease. The pericardium, he says, is in every respect analogous to a synovial bag; and by this alleged analogy of structure he accounts for the very frequent coexistence of pericarditis with articular inflammation. Like almost all the other favorite doctrines of M. Bouillaud, this one must be received with much reserve. It is quite *correct* in part, or to a certain extent; but most assuredly it is quite *incorrect* when carried to such "outrance," as he does. Another pet idea of his respecting inflammatory affections of the heart is, that in almost all cases of pericarditis there is co-existing endocarditis, or inflammation of the lining membrane of its cavities. There is much obscurity on this subject as yet; for hitherto it has not been examined by the more cautious pathologists of the French School, or by those of our own more phlegmatic and slow-going country, with very minute attention. M. Bouillaud is far too hasty an observer, and too rapid a reasoner, to carry much weight by himself. He certainly however deserves considerable credit for having directed the attention of physicians to the state of the endocardium, as well as of its analogue, the pericardium.* Whenever the endocardium is inflamed, there is induced a strong tendency in the blood within the heart's

* We recently observed the announcement of an English translation of M. Bouillaud's late work on Diseases of the Heart. We trust, for the sake of the translator's finances, that he does not propose to give the entire substance of M. B.'s very prolix volumes in an English dress. The book will never sell to pay the printer's expenses. Dr. Hope's excellent work, and the last published number of Dr. Copland's Dictionary, may quite supersede the French Treatise.

This timely caution may save the translator much expence, as well as annoyance. If he does persevere in his cavities to coagulate; and as there is usually a slight exsudation of lymph from the affected surface, we cannot be surprised that, in such cases, we usually meet with large and seemingly organised clots adhering to the walls of the ventricles, on dissection.

*Erysipelas* is another of those diseases, on which M. Bouillaud takes great pleasure in descanting; as it gives him a constant theme for exultation at the extraordinary success, which attends his practice. This may be at once presumed to consist in large and repeated bloodlettings. He acknowledges that many cases of this disease are accompanied with symptoms of typhoid and not of synochal fever; but, in spite of this, he insists upon treating all cases alike: "tous sont gueris rapidement par les saignées repetées."

The only purely nervous disease, mentioned in the Clinique, is chorea. His practice in this disease consists in the use of cold douche baths, the exhibition of musk and assafœtida in enemata, and the use of the Pilules de Meglin inwardly. He makes no allusion to the effects of steel in this disease.

It is certainly by far the most efficacious remedy, after the proper evacuation of the bowels by active purgatives. When once the state of the digestion is healthy, we should, without delay, commence the use of the carbonate of iron, and increase the doses, until a cure is effected.

---

Increase of Suicide in Paris, Poisoning from Colchicum, &c.

M. Charles Dupin, in his late discourse (unpublished), has alluded to the melancholy increase of this crime in the French metropolis. He attributes the fact to the unsettled condition of the public mind, and to the frequently recurring states of political excitement. The following table exhibits an almost regularly progressive increase during the last six years.

| | | |
|---|---|---|
| In 1830 | .... | 269 Suicides. |
| 1831 | .... | 377 |
| 1832 | .... | 369 |
| 1833 | .... | 338 |
| 1834 | . .. | 436 |
| 1835 | .... | 477 |

It is interesting to ascertain the age, at which the crime of suicide is most frequently committed. Annexed is a table, which affords some useful data to determine this point, and which also shews the relative proportion of suicides to the entire number of persons at different periods of life.

| Age. Years. | Suicides. | Population. |
|---|---|---|
| From 10 to 15 .. | 12 .. | 50,000 |
| 16 to 20 .. | 38 .. | 71,000 |
| 21 to 25 .. | 63 .. | 73,000 |
| 26 to 30 .. | 67 .. | 70,000 |
| 31 to 40 .. | 107 .. | 117,000 |
| 41 to 50 .. | 115 .. | 91,000 |
| 51 to 60 .. | 85 .. | 74,000 |
| 61 to 70 .. | 41 .. | 51,000 |
| 71 to 80 .. | 14 .. | 20,000 |
| 81 to 90 .. | 2 .. | 4,000 |

What a theme for reflection does this table present to the physician, and to the moralist; and not less to the political philosopher. How small is the number of those advanced in years, and labouring under the infirmities of decrepitude, or the pains and trials of protracted disease, who dare to snap the thread of life asunder; while in the ardent and impetuous season of youth, when health and bodily vigour most abound, and the capacities of enjoyment are most ample, the passions, those vultures of the mind, too often lead on the unhappy victims to self-destruction.

It is in manhood, however, from the age of 40 years to 50, that the suicidal mania attains its maximum of frequency. The animal energies are, it is true, still in full force, and the mental powers have, or, at least ought to have, reached the maturity of their powers; but it is to be remembered that perhaps at this period of life, more than at any other,

---

intention, he will do well to give a condensed and abridged digest of not only M. Bouillaud's, but of the other recent French pathologists' researches.

the mind is exposed to the disturbing influence of disappointed ambition, of domestic anxiety and distress, and of other causes of chagrin and disquietude; and that it no longer possesses that elasticity or resiliency of spirit, by which it relieved itself from vexing care in more youthful years.

The middle-aged man feels, when calamities overtake him, that he is less able, than he was wont to be, to struggle against them; and the mortification at the change of his circumstances, coupled with the slender hope of regaining his former position, is too apt to prey upon his mind, until he is driven on to commit suicide.

In a statistical note on England, published in a recent number of the Bibliotheque de Geneve, we observe it stated, that, in " that classic land of suicide," the crime has of late years very sensibly diminished. According to the bills of mortality in London, it appears that during the years 1834 and 1835, there were only 83 deaths from suicide, or not more than one in every 519 deaths; whereas in Paris the proportion is at present nearly that of one in every 100 deaths.—*Gazette des Hôpitaux.*

The number of cases of suicide reported in the French Medical Journals is very great. Scarcely a number of the Archives, or of the Annales d'Hygiene, is free from some melancholy details of murder or self-destruction. We have selected a few examples to illustrate the preceding remarks.

In the Archives Generales for May last, there is the account of a boy, 12 years old, who was found hanging in his bed-room. He had fastened his handkerchief to a nail in the wall, and having then passed the loop round his neck, he had swung himself off from a chair. When found, he was quite dead, although his feet rested on the floor. This boy was noted for " l'intelligence et la gentilesse dans ses jeux avec ses camarades." The only cause, which could be assigned for the dreadful act, appeared to be, that on the preceding day having accidentally broken his father's watch, he was shut up in his room, and nothing allowed him but dry bread, as a punishment for his carelessness.

Another case of suicide committed by a boy, eleven years of age, is narrated in the same Journal. It appears that he had left his father, by whom he had been reproved, in the fields, went home, put off his ordinary clothes, dressed himself in his holiday's suit, and then hung himself from the cross-beam of the bed. He had written on the wall with a piece of charcoal the following words :—" O sadieu de Francois Benjamin Ricard, qui s'est pendre atacher au Rido de sa mere."

It appears that a few months previously the uncle of this boy had hung himself. At his feet was found a bottle of holy water (eau benite, eau lustrale), and on the wall opposite to him he had marked three figures of the crucifix. The boy, following his example, had gone into his father's cellar, and had taken out a bottle of blessed water: it was found on the table of the bed-room.

These two cases occurred in Paris; the following one happened at Meaux. A youth, 13 years of age, only son of parents in easy circumstances, had been reprimanded and struck by his father for some offence. Next day he told his companions his intention to drown himself. " J'ai été frappé par mon pere, il ne recommencera plus ; je vais me jeter à l'eau." They laughed at this language; but unhappily the threat was not an idle one; for the boy that very afternoon did drown himself.

We are next told of two girls, the one eleven, and the other thirteen years old, who made attempts at suicide. The former had been threatened by her father with his displeasure, if she did not finish some task by a certain time. Afraid of not pleasing him, she became sad and unhappy, " et dès ce moment elle ne pensa plus qu'à mourir." She left her father's house, early one morning, and directed her steps towards the Quai St. Bernard, and there threw herself into the Seine. Fortunately the water was not deep, and some labourers having observed her, hurried to the spot and drew her out. In the other case too, it seems that the girl had had a quarrel with her mother. She also was saved, by some workmen having seen her throw herself into the river.

These are melancholy examples of the crime of suicide in early life. The following case presents a striking contrast, in respect of the age of the victim.

A man, 86 years old, who was in perfect possession of his reason, had made several attempts to put an end to his life, because he thought that men, however healthy and free from infirmities, ought not to exceed four-score years of age!! His companions had often tried to laugh him out of this strange notion; but in spite of all their reasonings with him, he at length effected his purpose by blowing his brains out.

The prevalence of suicide at the present time in France, and particularly in Paris, is truly frightful. The "jeunes gens," of that dissipated capital have, of late years, exhibited quite a suicidal phrenzy. Scarcely a day passes, but some fresh victim or victims of self-destruction are exposed at the Morgue; and every work, which has of recent years been published on the state of the French nation, teems with examples. The unsettled state of public affairs, the sad want of sound religious principles, and the wide diffusion of a most licentious and profligate literature, are no doubt the chief causes of this deplorable vice. It is not want, nor bodily suffering, nor mental distress, which has driven most of these unhappy wretches to launch themselves into eternity,

> "Unhouseled, unappointed, unanealed,

> With all their imperfections on their head;"

but in too many cases only a most daring defiance of the *vulgar* terrors of religion, or perhaps a most blinded ignorance, or unhappy disbelief of these very terrors.

In Mrs. Trollope's work on Paris and the Parisians, we read an account of two youths who went to one of the most fashionable restaurans, and ordered a most sumptuous dinner. After enjoying themselves with every delicacy, they summoned the maitre-d'hotel, and very coolly informed him that they had no money to give him; that for some weeks past they had been very badly off; and that, as they had made up their minds to commit suicide, they were resolved to have at least one capital dinner before leaving the world.

The restaurateur, not being satisfied with this story, was inclined at first to commit them into the hands of the police; but considering afterwards, we suppose, that this detention would not indemnify him for his loss, he allowed them to go. Next day these two youths were found dead in bed together!

---

### On Gastro-Malacia, or Gelatiniform Softening of the Mucous Coat of the Stomach and Intestines.

This pathological condition of the mucous membrane of the stomach and upper portion of the small intestines is, although not peculiar to early life, much more frequent in infants and young children, than in more advanced years. It seems to be, in many cases, not the mere result of preceding inflammatory action; but rather a disease "sui generis;" and to be most strictly and truly of idiopathic origin. The period of dentition, and more especially during the act of weaning, is that, when the morbid process of intestinal ramollissement is most apt to occur. It is certainly less common afterwards; and the disposition to it seems to be greatly less after the second year, than it is before.

Of 50 cases seen by Romberg, 6 only occurred later than the close of the second year.

As to the inducing cause of this disease, we are by no means provided with any very conclusive information. Romberg is inclined to ascribe it to some disturbance in the mutual relation of the salivary and of the gastric secretions, upon the cessation of the act of sucking; and he suggests therefore that, for some time after weaning, a child should always be fed from a glass, from which it sucks, and never with a spoon (!)

Other physicians have attributed this softening of the ventricular mucous coat, to the secretion of an acrid irritating gastric juice. But the occasional circumstance of portions of the mucous coat of the small intestines being similarly affected, at the same time as that of the stomach itself, is rather opposed to this interpretation.

Dr. Cammerer, of Stuttgart, in 1828, published an excellent work on this subject, wherein he suggested that the ramollissement of the mucous membrane might probably be owing to an interruption, or to some other disturbance, of the nervous influence of the par vagum, and sympathetic nerves on the intestines. In corroboration of this doctrine, he adduced the results of several experiments which he had performed on rabbits and some other animals; in which he had divided these nerves in the neck, and found that a genuine ramollissement of the mucous coat of the stomach and bowels had taken place.

His doctrine is, that these nerves are, in the first instance, sub-acutely inflamed; and that this inflammation induces a degree of paralysis of their energies; in consequence of which the still secreted gastric juice acts upon the coats of the enfeebled stomach, as it is well known to do after death in animals killed during the act of digestion. It must be confessed, that this hypothesis of Dr. Cammerer is purely conjectural. Neither he, nor any other pathologist, has been able to discover any distinct traces of inflammation of the nervi vagi, or of any other nerves; and therefore we cannot, says Dr. Droste, quite agree with the propriety of his therapeutic injunction—"that the great object of the physician is to subdue the inflammation of the nerves, and thus prevent the paralysis." The local bleedings, and the inunction of mercurial ointment may, so far from being of use, very probably increase the tendency to weakness, and softening of the predisposed tissues, if no inflammation exists.

The remedial means, however, which Dr. Cammerer has so strongly recommended to fulfil the other indication—to prevent the paralysis—meet with the warm approbation of our author. The remedy is the muriate of iron.* We shall adduce one case in which it appeared to exert most beneficial effects.

An infant, nine months old, became restless, feverish, and sleepless. The appetite ceased, and the stomach was irritable and sick, although the child seldom vomited. There was much thirst, occasional twitches of the facial muscles, cramps in the limbs; and the expression of the features indicated great internal uneasiness. No very appreciable cause could be assigned for this illness.

Repeated doses of calomel, sometimes alone, and sometimes combined with the flowers of zinc, were exhibited. The unfavourable state of things continued for several days; but then happily abated; and the child began to recover his appetite and liveliness. It was found, however, that almost invariably after taking any food, the child became restless, uneasy, began to cry, as if suffering from inward pain, and soon rejected by vomiting the contents of the stomach.

The author, Dr. Droste, now determined to administer the chalybeate salt, as he imagined that the train of symptoms depended upon an incipient softening of the mucous coat of the stomach. The formula, which he prescribed, was the following: ℞. Tinct. ferri Muriat. ℈j. Extract. Cort. Chinæ ℈j. Aquæ flor. Aurant. ℥ij. Syrupi Aurant. ℥i. Misce. A tea-spoonful of this mixture was given very frequently.

---

* Some of the German writers praise highly the sulphate of copper in the same sort of cases. We suppose that it is administered as an astringent and tonic, for the purpose of counteracting the morbid laxity of the mucous membrane.

All the unpleasant symptoms speedily abated, and the infant was soon restored to perfect health.

[The English reader will very properly question the accuracy of the diagnosis in this case. For our parts, we should have regarded it as one of simple weakness, or atony of the stomach, induced by a preceding feverish attack. The Germans, however, delight to apply all the subtleties of book-learning to the explanation of the most ordinary occurrences.]

That numerous cases of obstinate and fatal diarrhœa, in infants more especially, are dependent upon a ramollissement of the mucous intestinal coat, is a very generally received opinion among the German, and also many of the French pathologists. We find the following two cases, set down as examples of acute gastro-malacia, in a late number of Hufeland's Journal. They are reported by Dr. Durr, one of the chief physicians in Wurtemberg.

A child, nine months old, which had been reared without the breast, had for several weeks past been loosing flesh, in consequence of a general feverishness, troublesome cough, diarrhœa, thirst, and loss of appetite. The alvine evacuations were green and offensive. To these symptoms succeeded vomiting, pallor, and great anxiety of the countenance, heat and restlessness of the head, slight convulsions of the limbs, and spasmodic stiffness of the back.

Leeches, oily emulsions, purified argilla—[is this the Armenian bole of our pharmacy?—*Rev.*] the aqua oxymuriatica, &c. were used, but with no benefit. The infant died in the course of a very few days.

On dissection, the right side of the heart was found to be pale, and of a very soft consistence; the stomach was collapsed; its coats were pale, and of a marbled appearance; the left side of it was converted into a gelatinous pulp, and at one spot there was a perforation, as large as a hazel-nut.

A young infant, which had been reared with spoon-food, was distressed with purging of a green offensive water, with feverishness, loss of sleep, thirst, &c. An emulsion, containing half a drachm of the Argilla Depurata in an ounce and half of emulsion was ordered. The symptoms were mitigated in consequence; but, after the lapse of a week or so, they returned with increased severity, and were accompanied with aphthæ in the mouth and fauces. The same mixture, with the addition of one grain of extract of cicuta, was prescribed, and again succeeded.

These relapses of diarrhœa occurred repeatedly during the following two months: but ultimately the child recovered perfectly.—*Journal der Pratischen Heilkunde.*

We confess ourselves somewhat unable to form any just appreciation of the disease, which the German Journalists mention so often, by the name of Gastro-Malacia. Their descriptions are so very defective and confused, that all attempts to present a rational analysis of their writings are, to us at least, nearly impracticable. We shall, however, take this opportunity of briefly illustrating some of the most interesting particulars, regarding the softening of the intestinal mucous membrane.

Softening of the mucous membrane of the stomach and bowels may occur either from the operation of disease during life, or from the direct action of the gastric juice, immediately after the death of the animal.

In the healthy state, the mucous membrane of the alimentary canal is found to vary much in thickness and in consistence, at different parts of its extent. It is thickest and firmest in the duodenum and at the pylorus; and thinnest in the colon and at the fundus of the stomach. It becomes less and less thick and firm in the rectum, jejunum, and ileum. Where it is thickest, it may be torn from the submucous cellular tissue in shreds of from a quarter to half an inch in length; whereas in the colon and fundus of the stomach, it can be detached only in very small pieces. When the body is much emaciated, the mucous membrane of the whole extent of the bowels becomes so thin, that it breaks, whenever we attempt to separate it.

Softening of the mucous membrane from inflammation may occur at any portion of the intestinal canal. Dr. Carswell is of opinion, that this morbid change is most frequently observed at the termination of the ileum, and in different parts of the colon. But it may occur at any part; and in some rare cases, the whole extent of the tube, from the cardiac orifice of the stomach to the extremity of the rectum, may be affected with it.

There are various degrees of this morbid process. In the first degree, the mucous membrane is found to break, or tear across, whenever we attempt to raise it with the fingers or the forceps; in the second degree, the softening is so much greater that, by merely scraping it lightly with the edge of the scalpel, it is converted into a soft, opaque, creamy-looking pulp; and in the third or extreme degree, it is so loose and soft, that a stream of water is sufficient to detach it from its subjacent connexion. In this last stage, portions of the mucous membrane are found, on dissection, partially or entirely destroyed, and the submucous cellular tissue is laid bare in irregular or round patches of different sizes.

Softening of the intestinal mucous membrane is accompanied with various degrees of redness. The membrane is sometimes quite pale; at other times the redness may be confined to the softened parts, while the rest of the membrane is pale, or vice versà.

The redness may vary from a slight rose-red to a purple or dark brown hue. The pale inflammatory softening, of a grey, yellowish or milky aspect, is very common in phthisical subjects, in tubercular disease of the mesenteric glands, and indeed in all diseases attended with extreme emaciation.

Some authors have alleged that the inflammatory softening may affect all the tunics of the stomach and intestines, and may give rise to perforation of the tube at any part. Dr. Carswell disbelieves this statement. His words are: "softening of all the coats of the stomach or intestines, carried to the extent of perforation of these organs, does not arise in inflammation. It depends on the chemical action of the gastric juice."

There is one form of softening of the intestinal mucous membrane, which, from the alleged peculiarity of the symptoms attending it, and from the extreme rapidity of its progress, has attracted much notice from the continental pathologists. Cruveilhier has described it under the title of "maladie gastro-intestinale des enfans, avec desorganisation gelatiniforme."

This is no doubt the disease to which Dr. Droste in the preceding article gives the name of *gastro-malacia*, or die gallertartige magenerweichung im kindlichen alter.

According to Cruveilhier, the symptoms of this very violent and fatal disease are excessive thirst, urgent vomiting, and purging of very fetid, green stools. The child becomes exhausted, and cold on the surface; the pulse is slow and irregular; the infant is constantly restless and crying; drowsiness and oppression come on; and in from 24 to 48 hours a fatal collapse succeeds.

This affection has been not unfrequently mistaken for a violent cerebral attack, in consequence of the somnolence and screaming, the irregularity of the pulse, &c. &c. being common to both. But in the peculiar disease, now under consideration, the drowsiness is not deep or oppressive; the child is awakened by the slightest touch; the sensibility of the skin, moreover, is morbidly acute; and there is not the same tendency to delirium and cerebral confusion.

On dissection, the mucous membrane of the stomach, and generally of some part of the large and small intestines, is found reduced to a state of gelatiniform pulp, without perhaps any traces of inflammatory action.

M. Guersent also, one of the most experienced practitioners in children's diseases, has given a description of the gelatinous softening, as observed by him in the Hôpital des Enfans. This peculiar combination of vomiting and purging, he remarks, is most common between the third and fourth month, and the completion of teething. It usually commences with a diarrhœa of green evacuations; and, when this has lasted for some time, vomiting succeeds. The system is feverish; the infant restless and constantly moaning; and the prostration of strength extreme. It is seldom that we observe regular convulsions supervene; but there is usually a tendency to twitching of individual muscles, as of those of the face, neck, or extremities. The child generally remains quite conscious to the last—thus affording a very marked distinction from cerebral diseases, with which this affection has been frequently confounded. The duration of this affection, at least in its acute form, may vary from one to three or four days. Sometimes however it is of a more chronic character; there are frequent renewals and temporary cessation of the symptoms; the child's strength is gradually undermined; and at length it sinks, in perhaps a month or even a longer period, after the commencement of the mischief.

When the body is examined, the stomach and intestines are usually found pale, and almost transparent; as if they had been macerating, for a length of time, in water. Occasionally patches of inflammatory redness are discovered; but these are rare. It is however particularly to be observed that M. Guersent has met with a multitude of cases, where all the symptoms above enumerated were present, but in which he could detect no appearance of softening of the mucous membrane in any part of the gut.

The treatment, recommended by him and by Cruveilhier, consists in the use of demulcent drinks, emollient enemata, narcotic cataplasms and fomentations. Leeches and indeed all depletory means are positively injurious. Minute doses of opium are perhaps the most powerful and useful medicines. In chronic cases we may employ alteratives, astringents and tonics; but, in the acute form of the disease, there is no time to be lost, and we must have recourse at once to anodynes. It is not possible to lay down any general rules respecting the diet and management of the infant. In some cases it may be necessary to wean it immediately; at all events, it is always proper to give other food during the severity of the attack. Perhaps thin arrow-root, to which a small portion of brandy has been added, is the best. Whatever may be the real existing cause of this very fatal disease, we cannot attribute it to mere inflammatory action—at least antiphlogistic remedies are certainly the worst that can be used. The softening of the mucous membrane appears rather to be truly idiopathic.

The form of softening of the intestinal mucous membrane, now described, is not peculiar to infants. It is occasionally observed in youths and adults; but in the former more frequently than in the latter. We have seen it in several cases in young females, who had been ailing for some time previously under some obscure abdominal or thoracic affection, and had become much reduced and emaciated. An indiscretion in diet, or exposure to cold, or even a sudden mental emotion, has brought on an alarming degree of diarrhœa, and the patient has sunk in 16 or 24 hours. On dissection, some part of the intestinal canal has been found softened and perhaps so much disorganised, as even to permit the finger to perforate its walls without much difficulty. We believe that nothing can be done to save such cases. At least opium and other narcotics are the only remedies, which hold out any prospect of benefit.

As for the recommendation of the preparations of iron or of copper in the gelatiniform softening of the stomach by Dr. Droste and other German writers, we hold the practice very cheap. It is founded on far too a chemical or mechanical an idea, to promise any good in controlling a vital action. Iron and copper may harden a piece of leather; but we much doubt whether they can restore a softened stomach to its healthy firmness.

We shall now discuss very briefly that other form of softening of the stomach and intestines, which is caused by the direct chemical action of the gastric juice, immediately after the death of the animal. This very singular phenomenon was unknown to pathologists, before the publication of John Hunter's celebrated paper, " On the Digestion of the Stomach after death," in the Philosophical Transactions. His experiments and observations were repeated and confirmed by Spallanzani, Dr. Adams, and Mr. Allan Burns of Glasgow. It was the latter gentleman who, we believe, discovered this solution of some portion of the stomach in persons emaciated from chronic disease, as well as in those who had died suddenly, or in animals killed during a state of perfect health.

The opinions of Hunter were at first received in France with great reluctance; and numerous conjectures were brought forward to account for the solution of the coats of the stomach. It was generally regarded not as the effect of the gastric juice; but as a serious lesion caused either by long-continued disease, or by a rapidly-excited high degree of local irritation, induced somehow or other, and ending in the destruction and disorganization of the part. Even some of the leading pathologists in the present day, such as Cruveilhier and Louis, consider the appearances, attributed by Hunter to the solvent power of the gastric juice, as a peculiar diseased alteration of the affected tissues, hitherto overlooked by anatomists.

M. Louis, in his Recherches Anatom. Pathol. 1826, has described minutely the *symptoms*, and morbid appearances, that he observed in a multitude of cases in which the mucous membrane of the stomach was found to be more or less softened. He regards this disease as depending upon a peculiar irritation of the mucous membrane, the symptoms of which are often very obscure and uncertain.

Cruveilhier also has come to the same conclusion respecting this disease, more especially as it is found in infants and young children. Dr. J. Gairdner, in a very able paper published in one of the early volumes of the Edin. Medico-Chir. Transactions, expressed his sentiments on this subject to the following effect:—that the softening and perforation of the alimentary canal, frequently observed in the bodies of infants, are produced after death by the action of the fluids contained in the stomach and intestines; that these lesions probably occur in some cases without any previous disease of the parts; but that they also take place in consequence of a peculiar disease of the canal, whereby portions of it are rendered more easily soluble by the action of its contents.

Dr. Carswell also, certainly one of the highest authorities on every thing connected with pathological anatomy, has come to the conclusion that softening, erosion, and perforation of the stomach, intestines, œsophagus, diaphragm, &c. have been met with, of every possible degree, extent and form, whatever may have been the nature of the disease by which they had been preceded; and that these lesions have been produced by the solvent action of the gastric juice and intestinal fluids. "It is an interesting fact," he observes, "that in almost all the cases of softening (gelatiniform softening) with perforation, which have been published, the large intestines, situated in the epigastric or left hypochondriac region, were exclusively the seat of one or both of these lesions. Such also has been the result of our own observations in all the cases of softening of the intestines, which we have examined in the dead body. The locality of this lesion and its physical characters being precisely the same in man, as those observed in animals (killed soon after food has been taken), and having traced it in both to the mediate or immediate influence of the gastric acid, we must necessarily regard it in the former, as it has been shewn to be in the latter, case, a *post-mortem* alteration." In a subsequent part of his memoir, he remarks "that inflammation produces quite a different kind of softening of the mucous membrane, from that which we have described. The mucous membrane softened by inflammation, instead of being transparent, is more or less opaque, and, even when it is completely disorganised, it resembles a mixture of flour and water, or of milk, rather than an albuminous, or a gelatinous fluid. Such is, in fact, the principal character of inflammatory softening of mucous membrane, in whatever organ it occurs; whereas the *transparent gelatiniform softening* is never observed, except where the chemical agent is formed by which it is produced—viz. in the alimentary canal, and in some of the neighbouring organs." Dr. C. alludes also to the frequently total absence of all traces of inflammation in the abdominal viscera, such as redness, effusion, lymph, &c. when not only the stomach and intestines, but even the diaphragm, spleen, and liver, have been found affected with this process of disorganising softening.

We have not space to allude to those experiments on the lower animals, where they have killed soon after taking food; and to those observations on the human subject, where death has occurred suddenly after a meal, and which place beyond all doubt the perfect accuracy of the original propositions laid down in Hunter's paper in the Philosophical Transactions. Suffice it to say that our readers will do well to consult the admirable writings of Dr. Carswell.

Whether this able pathologist is correct, in considering all cases of gelatiniform softening of the stomach and intestines as depending upon the action of the gastric juice, appears to us more than doubtful.

That this morbid process takes place without preceding inflammation is admitted by all the best writers; but whether it is always the effect of the solvent power of the intestinal juices, or occasionally the result of an idiopathic and vital process, is not definitely settled. Andral leans to the latter opinion; and thus confirms the views of Cruveilheir and Guersent, and of the German anatomists. All these authors agree in describing the symptoms of this affection as of a choleriform character—diarrhœa, vomiting, general anxiety and restlessness, coldness of the extremities, and lastly convulsions and death. The attacks are most frequent during dentition, and more especially at the period of weaning.

The morbid changes, found on dissection, vary in degree from a mere diminution in the consistency and cohesion of the affected membrane to a state of complete and almost putrid gelatinous softening, accompanied with patches here and there of extravasated blood.

Whether this morbid softening may so affect all the coats of the stomach or bowels as to cause perforation, and thus give occasion to the escape of their contents into the cavity of the abdomen, *without* the simultaneous solvent action of the gastric juice, may be fairly questioned. Dr. Carswell, as already stated, is opposed to this view; and perhaps quite correctly.

The propriety of arresting severe attacks of diarrhœa, in teething children, is a question of too great extent to be discussed here. For one case, which will be benefitted by muriate of iron, sulphate of copper, and such like remedies, ninety-nine require the use of mercurial alteratives, antacids and mild purgatives. By far the best plan of treatment, as a general rule, will be found to administer from three to five grains of the hydrargyrum cum cretâ, alone or in combination with the compound chalk powder, and to follow this up with repeated doses of such a mixture as the following:—

℞. Magnes. calcinatæ, ℈j.

Carbon. calcis preparat. gr. xv.

Syrupi Rhei, ℥ss.

Aquæ anethi, ℥iss.

Spir. ammoniæ fœtid. ♏xij.

Vini ipecac. ♏xx.

Tinct. hyosciami, ♏xij. Misce.

A dessert-spoonful for a dose every hour or two. When the diarrhœa has ceased for some time, it will usually be advantageous to exhibit a small dose of castor oil, for the purpose of carrying off any offensive matter lodged in the bowels.

We must always be very cautious of suddenly and completely checking diarrhœa in infants with opiates. Many a case of confirmed hydrocephalus has followed such practice.

---

### Cursory Remarks on Hydrocephalus Acutus, with Cases.

The number of cerebral diseases to which infants and young children are subject, is by no means large. Genuine apoplexy is rarely or never met with in them; epilepsy is of very uncommon occurrence; and so is chorea and tetanus: by far the most frequent head-affections are hydrocephalus (or as it ought rather to be called 'meningitis,' or 'meningo-cephalitis') and convulsions. None of the descriptions, which authors have given of the former of these diseases, will be found faithfully to represent and portray the actual phenomena, as seen at the bed-side of the patient; and we do not yet possess a work which we can confidently recommend to the young practitioner, to guide him in the diagnosis and treatment of hydrocephalus. Dr. Whytt attempted to arrange the symptoms of the disease under a threefold division; he has insisted particularly on the different characters of the pulse, as indicative of the three stages. Experience however does not confirm, on all occasions, the accuracy of his description.

Dr. Coindet thought that the moaning cry of the little patient, in genuine hydrocephalus, is quite characteristic of this affection. This is a fanciful idea,—and not warranted by practice; for most certainly the "cris hydrocephaliques," described so minutely by this author, are heard as often in other diseases. The statement which M. Serres has made "that the central parts of the brain, viz.—the corpus callosum, septum lucidum and fornix, are invariably found on dissection to be unaffected in those cases of hydrocephalus, in which the sensibility of the patients has been preternaturally exalted, and has not been impaired in the last stage," may perhaps be true in a multitude of instances; but assuredly it is not in all.

Every practical physician is well aware, that this most formidable disease, at least its first stage, is often simulated by certain abdominal affections; and that hitherto no decisively diagnostic symptoms have been pointed out, by any of the numerous authors, who have written its history. In this state of uncertainty, it will be prudent therefore to have our attention directed, in all suspicious cases, to the state of the head; for it is better to treat many abdominal affections, as if they were of cerebral origin, than to commit the contrary error. Many cases of typhus very closely resemble the course of genuine hydrocephalus; and indeed it is not a very unfrequent occurrence that encephalic effusion supervenes to a protracted typhoid or nervous fever, in certain delicate constitutions. It is however worthy of notice that genuine typhus seldom attacks children at that period of life, when they are most subject to the attacks of hydrocephalus, viz—from two to six years of age. The following series of cases cannot fail to be valuable, as teaching by example some of the most prominent features of this very formidable disease.

CASE 1.—*Vomiting at the commencement—pulse becoming quicker and quicker—disturbance of the motility—death on the 10th day.*

S. M., two years old, had been ailing, for three weeks, from cough and diarrhœa. When she was brought into the hospital, on the 23rd of May, the existing symptoms were fretfulness, crying whenever she was touched or moved; cough, but no pectoral stethoscopic irregularity; pulse 108. On the evening of the 26th, she vomited; the face was observed to have frequent alternations of redness and paleness—pulse 96: some leeches were applied behind the ear. Next day there was strabismus and confusion, so that she did not know her mother; a stiffness in both arms; bowels not purged for two or three days; pulse 104 and irregular. The stiffness of the arms increased and extended to the muscles of the neck and of the trunk; pulse (on the 30th) more rapid, having risen up to 120. On the 1st of June there seemed to be amaurosis with the strabismus, and also twitchings of the muscles of the jaw and cheeks came on;—pulse 132. 3rd—extreme pallor of the skin; quite insensible; pupils dilated; extremities cold; pulse very frequent. On the 6th she died, without having suffered any convulsions.

*Dissection.*—Four ounces of serum flowed out on lifting off the calvarium. The arachnoid coat was pale on the upper surface of the brain; and at the base, especially about the fossa silvii, there was a subarachnoid whitish sero-purulent effusion, and also an appearance of pale-coloured granulations of the size of millet seeds. The medullary substance of the brain was generally softened; the fornix and corpus callosum in particular were very easily lacerable.

*Reflections.*—So suspicious a symptom is vomiting in infants, when it occurs without any appreciable cause, that we should immediately, under such circumstances, direct our attention to the head. In the above case, the alternations of flushing and pallidity of the face at the commencement, and subsequently the fixed pallor of the features, were very conspicuous symptoms. The pulse became more and more frequent, from the commencement to the fatal issue of the case.

Case 2. *No vomiting at the commencement—Stupor interrupted by two days of agitation—Death on the 13th day—Subarachnoid effusion.* A girl, aged 2½, had been ailing for five days before she was brought to the hospital. The symptoms on the 23d of June were redness of the face; the eyes were kept closed; the child was fretful and sleepy; the breathing was sighing; there was much thirst but no vomiting or purging; pulse 80.

On the next day the pupils were observed considerably dilated; the other symptoms as before; pulse 92.

25th. Stupor; alternate flushings and pallor of the face; pulse 96; pupils contracted; leeches and calomel.

26th. Pupils dilated; conjunctivæ red; pulse 128.

27th. Great restlessness; pulse 126.

28th. Stupor, interrupted with occasional moaning; deglutition difficult; pulse 104.

29th. Stupor; dilated pupils; amaurosis and strabismus; pulse very frequent. Death in the evening.

*Dissection.* Five or six ounces of serum flowed from the brain. The arachnoid was pale on the upper surface; and there was a serous subarachnoid effusion at the base of the brain, chiefly around the two fossæ silvii—no trace of pus anywhere—substance of the brain firm and not more vascular than usual—ventricles dilated—corpus callosum and fornix easily lacerable.

Case 3. *Stupor continuing during the greater part of the disease—Death on 8th day—Serous effusion.*—A girl, aged 3, having been indisposed for four days, became unusually heavy and drowsy; she uttered shrill cries; and vomited occasionally; there was slight cough; and the pulse was 104. She continued in this state for three days; the somnolence was then more and more complete; and she could be roused only with difficulty;—pulse 120. In two days more she died without having experienced any convulsions.

*Dissection.* A slight purulent serosity was found between the junction of the optic nerves, and the crura cerebri. The arachnoid coat was pale on the upper surface of the brain. The cerebral substance was rather soft, and but little injected with blood: the corpus callosum and fornix were easily lacerable.

Case 4. *Hooping-cough—Stupor—Strabismus—Contractions of the limbs—Death on 10th day. Subarachnoid effusion, with granulations.* A girl, aged 3½, who was labouring under hooping-cough, was suddenly seized with drowsiness, strabismus, and other symptoms of cerebral disturbance; pulse 112.—There were frequent changes of the colour of the face, it being at one time pale, and at another time much flushed. For four days, the child continued in this state of oppression, not recognising any one, but lying nearly insensible; the pupils had become dilated; the pulse was still very frequent. On the 5th day the countenance was haggard, the eyes were open, amaurotic, dilated, and squinting; the child lay insensible; the muscles of the neck were so stiff and permanently extended, that the body might be raised, by lifting only the head from the pillow;—pulse 132. She occasionally coughed, but never with any hooping noise.

No change took place in her condition for other two days, she being all the time comatose. She died on the evening of the 7th day from the commencement, without having suffered any convulsions.

*Dissection.* The arachnoid membrane, on the upper surface of the brain, was highly injected; towards the base of the brain, it was infiltrated with a limpid serum, and studded with numerous whitish, semi-transparent, hard granulations, of the size of a pin's head. These were most numerous about the fossæ Silvii, and between the adjoining convolutions. The cerebral substance was softened throughout, and the cortical was so soft that at some parts it readily separated along with the arachnoid, when this was pulled off. About four ounces of serum were found in the ventricles. The substance of the cerebellum was very pale.

CASE 5. *Death 10th day.—Effusion.* A girl, aged 3, after a few days' indisposition, was reported to be drowsy, to vomit and cough occasionally; the skin was hot, and the pulse 96. Two days subsequently she had a convulsion; the eyes squinted, the pupils were dilated, the face was flushed, pulse 124. Though heavy and dull, she was sufficiently sensible to answer, when spoken to. On the 6th day, there were occasional convulsive twitches of the muscles of the face and of the limbs; the child moaned constantly; the pupils were still dilated. On the 8th she died.

*Dissection.* The arachnoid, on the upper surface of the brain, was very dry, and highly injected. There was partial serous infiltration at the base, especially around the larger arterial and venous trunks. The cortical substance came away along with the arachnoid; the medullary substance was firm and not injected. About 6 ounces of serum were found in the ventricles.

CASE 6.—A girl, aged seven, had been ill for a fortnight, when she was brought for advice. She then complained of headache, pain at the epigastrium, but there was no vomiting, nor diarrhœa. She was feverish, and had a troublesome cough. In spite of the remedies employed, the headache continued, and was attended with considerable drowsiness; the patient was unwilling to anwer questions, or to be in any way disturbed; the face was flushed, the eyes kept closed; tongue white—pulse variable, being 116 on one day; and only 96 the next day. Repeated leechings, and doses of calomel were employed. She became insensible; the pupils were dilated; she was occasionally convulsed; and died eight days after she was taken for advice.

*Dissection.* The arachnoid on the summit of brain was dry; at the base, and upon the cerebellum there was subarachnoid seropurulent effusion; the cortical substance came away adhering to the arachnoid, when this was pulled off. The medullary substance of the brain generally was very soft; and the lateral ventricles were much dilated.

CASE 7.—A girl, aged 8, was suddenly seized with vomiting, and intense headache; the face was distorted by the pain; the eyebrows contracted, the eyes bleared, the conjunctivæ injected. For several days this severe cephalalgia continued; the tongue was white and feverish; there was considerable nausea; the bowels were constipated; the pulse 96. Leeches, derivatives and purgatives were employed; but the suffering in the head did not abate; the child lay moaning constantly, and screamed, whenever the head was moved, or even the hair was touched. A short attack of convulsions supervened; the muscles of the neck became stiff, and the head was drawn back forcibly. The little patient was perfectly sensible, and answered all questions; but shewed great uneasiness, when she was in any way moved, or disturbed. The pulse was variable, from 95 to 98; then it rose to 116, and fell afterwards to 104. The headache had now lasted for nine days without intermission, or abatement: the pain must have been very severe, as the features were almost always grimaced. The abdomen became exceedingly tender, and the pulse rose to 128; she was restless and agitated, but never oppressed, nor insensible; and she continued to answer questions till within a few hours of her death, which took place on the 16th day from the commencement of her illness. There had not been either delirium, convulsions, or coma at any period. The death took place, sooner than was expected by the medical attendant.

*Dissection.* On the upper surface of the brain, the arachnoid was healthy; the pia mater was highly injected. At the base, especially between the optic nerves and the crura cerebri, in the fissures of Silvius, and around the basilar and communicating arteries, the arachnoid membrane was opaque and opaline, and lined on its inner face with a greenish-yellow puriform coating. The substance of the brain was healthy and firm. The lateral ventricles were considerably dilated, but they did not contain much serum.

Case 8.—*Remarkable Stupor, during the whole course of the Attack.* A girl, aged 6, had laboured under infantile remittent fever for about a week. She was then seized with vomiting, and became flushed in the face, and very drowsy. She put her hand fequently to her head, and seemed to suffer much pain there—pulse 76. There was little change in the symptoms, till the day of her death, which took place on the 10th day, after the attack; she lay motionless and oppressed all the time; but could answer questions, when urged upon her; the eyes were kept closed; strabismus occurred the day before her death; and strong convulsions also preceded it. For the four previous days, she was insensible; and the pulse had risen to 120, and even higher sometimes. The alternate flushings and pallor of the countenance were observable during the early part of the disease.

*Dissection.* Serous subarachnoid effusion was found at the base of the brain. The arachnoid membrane was healthy at the upper surface. The cerebral substance was generally soft; and the "parties centrales" were pulpy. The ventricles were dilated, and contained about 2 oz. of serum. Two hard yellow tubercles were found in the left anterior and in the right posterior lobes. Several tubercles also were discovered in the substance of the cerebellum.

Case 9.—*Death 39th day.* A boy aged 4, of a scrofulous habit, suddenly became fretful, dull, and unwilling to leave his bed. He did not however complain of pain anywhere; and his pulse was quite natural. The stomach became irritable a few days after; and he vomited his food occasionally. The face was every now and then very pale; he frequently moaned, and became unusually drowsy. The pulse was 100. There was little change in the symptoms for about a week. He was still sleepy, irritable when moved or disturbed, and moaning at other times. Now and then he vomited; and the pulse varied from 100 to 120. The drowsiness suddenly increased to a state of complete stupor; the face was alternately flushed and pallid; the eyes were kept obstinately closed; the pupils however were neither dilated nor contracted, and were quite moveable—strabismus and rolling of the eye came on; and, two days afterwards, some contraction of the left arm was observed; the fingers were kept firmly clenched; the breath became fetid, the pulse rose to 130; and after a few convulsions he died, at the end of the fifth week.

*Dissection.* A patch, an inch square, of blood was found between the bone and the dura mater, over the left middle meningeal artery. The arachnoid was healthy on upper surface of brain; and there was no infiltration, but at the base, especially between the optic nerves and the crura cerebri; and at the fossæ Silvii, this membrane was opaline, and presented several yellowish grey tuberculous particles; the adjacent cortical substance was very soft, and the medullary substance was redder than natural. The ventricles were dilated, and contained about 6 oz. of serum in their cavities.

Case 10.—*Cured.* A girl aged 5½, after several days anomalous indisposition, began to complain of her head; she vomited occasionally; was feverish, moaned much, was very drowsy; the bowels were confined:—she had voided a lumbricus. She was affected in this manner for six or eight days; and the report states, that then the face was pale, that she frequently pressed her hand against her head, that the pulse was 136; but that there was no vomiting, nor diarrhœa.

Next day, she was exceedingly restless and agitated; she frequently started screaming; the eyes were fixed; the pupils dilated; she did not answer questions, although she seemed to understand what was said:—face alternately flushed and pale—pulse 120. For several days these alarming symptoms, although less severe, continued. The prognosis was therefore very unfavourable. Very unexpectedly, in the course of a day or two, she began to recognize her mother, but she did not answer any questions put to her; and so insensible was she to external impressions, that the flies crept over her face, quite undisturbed. Notwithstanding these inauspicious symptoms, a decided improvement may be dated to have taken place, on the 11th or 12th day of the disease. The pulse retained, for some time, a great frequency, varying from 118 to 130.—*Archives Generales.*

*General Remarks.*

The preceding cases, although very imperfectly reported, will be found to illustrate some of the most remarkable phenomena of that most insidious and most dangerous disease, Hydrocephalus acutus. The great obscurity of its approach is well known to all experienced physicians. The child usually has been ailing somewhat, for a week or perhaps longer, before any serious symptom is observed. He has lost his usual cheerfulness; refuses to play; wishes to lie down, or to rest his head on the nurse's lap; and there is generally an irritability of temper, which was not noticed before. The sleep is disturbed; the little patient wakes often crying, or at least moaning; he is thirsty, but has little appetite for his usual food. He dislikes any strong light; and is offended by loud noise. In some cases, a tendency to slight convulsive twitches of the face, neck, or hands, has been remarked in the precursory stage of the disease.

Whenever such a train of symptoms is present, the prudent physician will have his attention directed to the state of the head. As a matter of course, he will examine into the condition of the thoracic and abdominal viscera; and very often some of these will be found more or less disordered. Still the amount of such disorder will, in all probability, not be sufficient to account for the great constitutional disturbance; and as the encephelon may certainly be regarded as the part of the organization in a young child, which is the most weak and liable to diseased action, it will be prudent at once to take measures to prevent, at least, the accession of more alarming mischief. Briskly purging with calomel and jalap, followed by the repeated use of aperative-diuretic medicines, leeches behind the ears, and cold applications to the head, may save the life of many a child, if resorted to in sufficient time. The gums should be examined, and freely scarified, if there is any tenderness, or if the child grinds his teeth.

There are two or three particular symptoms, which, when they occur in a child without a very obvious reason, should always excite the prudent apprehension of the medical attendant. One of these is the occasional vomiting of the food. The stomach is usually irritable in the precursory and early stages of hydrocephalus; and hence it has been frequently observed, that nausea and retching are present for several days, before the accession of the febrile and more alarming symptoms. It will be wise, therefore, on all occasions, to attend to the occurrence of what may be termed idiopathic vomiting in children—we call it *idiopathic*, because there is usually no very obvious cause for its coming on.

Another of the symptoms, alluded to above, is an accelerated state of the pulse; continuing so steadily, and without intermission or abatement, day after day. Whenever the pulse keeps, for a considerable length of time, above its natural standard in young children, we have strong reason to suspect that some internal mischief is going on. It is, however, to be remarked, that in some cases of hydrocephalus, the pulse is unusually slow during the precursory stage. This is certainly quite true, and is a fact which should not be lost sight of by the physician; but by far the most common occurrence is to find the pulse considerably and steadily quickened—ranging from 120 to 140, or even 160.

In short, any remarkable deviation from the natural frequency of a child's pulse may well excite some degree of alarm. In the treatment of hydrocephalus, the superior sagacity of the experienced practitioner is shewn not so much in the greater activity or variety of the means which he employs, as in the administration of them in due time. When once either softening of the brain's substance, or effusion on its surface, or into its cavities has taken place, it is very questionable whether the medical art can be of any avail in arresting the mischief. The aim, therefore, of the physician ought to be, to *prevent*, not to hope to *cure*, hydrocephalus acutus.—*Rev.*

---

### Cases of Croup, very actively and successfully treated.

Case 1.—A girl, seven years of age, had been affected with tinea capitis for upwards of twelve months. After an exposure to damp and cold, this cutaneous affection suddenly dried up, and cough, difficulty of breathing, and feverishness succeeded. The paroxysms of the cough became more and more alarmingly severe, and were accompanied with the peculiar shrill metallic crowing of cynanche trachealis. The pulse at this time was rapid, soft and compressible. Eight leeches were immediately applied to the throat, and a grain of calomel was given every two hours, along with half a tea-spoonful of a four ounce mixture, containing 3 grains of tartar-emetic, and 4 grains of extract of henbane. The tartar emetic ointment was also rubbed in upon the scalp every four hours.

On the following day, the 10th of November, a spoonful of an emetic mixture containing tartar emetic, ipecacuan and oxymel of squills was ordered to be given very frequently, until copious vomiting was excited; and then, when this was effected, two grains of calomel were ordered to be exhibited every two hours, a small blister to be applied to the chest, and the strong mercurial ointment blended with some powdered camphor and opium to be applied round the neck.

11th. No vomiting has been excited, but the child has repeatedly expectorated a quantity of thick and very viscid phlegm. She is in danger of being suffocated, whenever the head is low. The emetic mixture was ordered to be given every second hour, and the two grains of calomel every intermediate hour:—the throat to be well anointed with an ointment consisting of an ounce of saturnine ointment, with which was mixed a scruple of camphor, and the same quantity of opium, and also of carbonate of ammonia.

The cough became, towards the afternoon, looser and more free, and a quantity of exceedingly viscid mucus was repeatedly expectorated.

13th. Several large pieces of membranous phlegm were brought away by coughing. One of these, two inches long, had quite the circular form of the trachea, and even retained the impressions of its cartilaginous rings. The respiration became freer, the pulse slower, and the sputa began to exhibit a yellowish and red aspect. The medicines were ordered to be repeated at longer intervals.

15th. The child was much better. A mixture containing the extract of polygala senega, tartrate of antimony, syrup of althœa, extract of hyosciamus, and fennel-water was prescribed; and two grains of calomel were ordered to be given every evening.

17th. The sputa are thick and of a yellow colour: the voice is still scarcely to be heard. A decoction of the lichen islandicus along with the senega root, and a few drops of hydrocyanic acid were prescribed. These medicines were continued for a week, and the patient was declared to be quite recovered.

During the attack, 82 grains of calomel, and two drachms and a half of ipecacuan, and nearly half a drachm of tartar emetic had been administered.

*Case* 2.—Dr. Caspar was summoned to visit a young child 3½ years of age, suffering from a severe attack of croup. Four leeches were immediately applied to the throat; and one grain of calomel, and a dose of antimonial mixture were ordered to be given every two hours. Next day, the disease was worse; the cough being more severe, the dyspnœa more alarming, and the peculiar crowing sound more shrill and loud. The calomel was ordered to be given as before, and the dose of the antimonial tartrate to be increased. Mercurial ointment mixed with camphor and opium was smeared all round the throat and neck.

On the 3rd day (23rd of Nov.) the symptoms, although still very alarming, were somewhat mitigated. The hydrocyanic acid was given repeatedly in small doses; three leeches were applied to the sternum; and the calomel was exhibited at longer intervals.

On the 26th, the skin of the face and breast was covered with a measly rash. The eruption spread over the whole body, and the tracheal symptoms threatened to return in all their former severity. Fortunately the little patient got through the attack well, and gradually recovered his health and strength. The aphonia however continued for a long time, and appeared at length to yield to the use of a third part of a drop of cajeput oil given with sugar, night and morning, for some time.

The author has recorded a third case, treated in a similar manner, and with equal success. He alludes to the very large doses of emetic medicines which must be exhibited in this formidable disease, for the purpose of exciting vomiting. The first case affords a forcible illustration of this precept. He has much reliance, he tells us, in the external application of the mercurial ointment, to which some opium and camphor, and sometimes also carbonate of ammonia, are added, as a means of subduing inflammation. He has recourse to it, with much advantage, in cynanche tonsillaris, and in many other forms of inflammatory action.

To relieve the hoarseness and remove the dregs, as it were, of the disease, the decoction of lichen islandicus and of the polygala senega root, in conjunction with small doses of hydrocyanic acid, has been very useful in his practice. — *Wochenscrift für das gesammte Heilkunde.*

The therapeutic directions in the preceding remarks are exceedingly good, and have a much more practical character, than most of the articles in the German Journals can lay claim to.—*Rev.*

---

Peculiar Spasmodic Affection of the Thumb in Writing, Drawing, etc.

The gentleman, the subject of this most annoying complaint, had up to his 27th year been able to write and otherwise to use his right hand with the greatest ease. He was much given to drawing and painting. In 1801 he began to observe that he could not hold his pencil with steadiness, when engaged in any fine sketch. This annoyance increased so much, that he was obliged to give up his favourite amusement.

The thumb was affected with involuntary twitches; when these ceased, it pressed with so much firmness upon the fore-finger, that any attempt to draw or write was most troublesome and even painful. He experienced however no difficulty in grasping or holding any object with his right hand; but in all more delicate movements, in which the co-operation of the thumb is necessary, there was embarrassment and unsteadiness. In attempting to play on the harpsichord, he could not prevent the thumb from continually striking the notes, for it was in constant motion from right to left. The affection was regarded by some as of a merely nervous, by others as of a gouty character. All sorts of remedies were tried; but none seemed to have the slightest effect. For upwards of five years the patient was obliged to write with his left hand; and then, most unfortunately, his left thumb began to be similarly affected. He now heard of a fellow-sufferer having derived much benefit from the use of a ring put round the fore-finger, sufficiently large to allow a pen to remain between it and the finger, and tight enough to confine it in its place. With the aid of this simple contrivance he was again enabled to write with his right hand, being now in some measure independent of the co-operation of the thumb.—*Medizin: Zeitung.*

---

### Idiopathic Tetanus and Trismus connected with Ramollissement of the Spinal Cord.

A young man, soon after his admission into the army, found one evening that he could not chew his food, as usual, and that his neck and back were stiff, upon any attempt to move them. Next day, he could not open his mouth; the masseter and sterno-mastoid muscles were rigid and hard; and there was an expression of spasmodic laughter on the countenance. A solution of tartar emetic was ordered to be given at short intervals, and the patient was confined to bed. His sensorium was quite clear and undisturbed; the pulse was natural, and his appetite good. He was cupped very freely on the back of the neck, vomited with tartrate of antimony, and had a large blister applied over the nucha. In spite however of these means, the rigidity of the muscles extended and increased, so that all motion was impracticable. A very large detraction of blood by cupping was taken from the spine, and afterwards the patient was put into a warm bath, in which an ounce of caustic potass had been dissolved.

On the 26th (the third day of the disease) the cupping and alkaline bath were repeated; an antimonial mixture containing some laudanum was administered every two hours; frictions with equal parts of mercurial ointment and volatile liniment along the back were employed, and the blistered surface was dressed with one grain of muriate of morphia. This treatment seemed at first to have had some effect in moderating the tonic spasm; for the patient was able to open his mouth, so as to admit the introduction of one finger. Towards evening however, the contraction of the dorsal muscles amounted to frequent convulsions; and often a complete state of opisthotonos came on. No change occurred in the following three days, although the very active treatment indicated above was most perseveringly continued.

On the 29th, the cramps had increased in frequency and in severity, and the general rigidity also was greater than hitherto; the pupils of the eyes were small and immoveable; the abdomen was hard and stretched; and the patient was exhausted with alternate shiverings and perspiration. The tartrate of antimony and the muriate of morphia were now tried in the endermic method; two dozen of leeches were applied along the spine; two grains of calomel and a quarter of a grain of opium were administered every second hour; and the alkaline baths were continued. The frictions also with the volatile mercurial liniment were persevered in.

No favourable change however took place; and accordingly, on the 31st, two eschars, each six inches long, were made with the actual cautery along the sides of the cervical vertebræ. But no benefit was obtained; and at length the patient died apoplectic on the morning of the 2nd of November.

*Dissection.*—The veins and sinuses of the encephalon were gorged with blood. On opening the spinal canal, the vessels of the pia mater were found highly injected; and the spinal marrow itself in two places—the one between the 4th and 7th cervical vertebræ, and the other between the 3rd and 6th dorsal vertebræ—was so much softened that it resembled curdled milk, more than any other substance. The roots of those nerves, which arose from these altered places, were also affected with extreme ramollissement. The mucous coat of the stomach was softened, loose, and readily tore across in attempting to lift it up.—*Medizinische Zeitung für Heilkunde in Preussen.*

---

### Case of Spina Bifida, with Practical Remarks on this Malformation.

The child was fifteen weeks old, when examined. The head was not unusually large, but the sutures and fontanelles were more open than in healthy infants. The rest of the body was normally formed, with the exception of the lower part of the spine; where a large swelling was situated over the lower lumbar and the upper sacral vertebræ. It was of the size of an apple at birth, but since that period it had increased so much, that now it measured upwards of eleven inches round its base, and projected nearly six inches above the surrounding integuments. The skin was tense, and exhibited a fine network of minute blood-vessels on its surface. There had been an excoriation at one part, which remained open for several weeks, but was now healed. A distinct fluctuation was readily perceptible on examination. The swelling was not diminished in size by pressure made upon it. The health of the child did not seem much affected, with the exception perhaps of its sleep being always restless and disturbed.

A small incision was made in the lower half of the swelling, and between three and four ounces of a fluid, which was at first slightly reddened and afterwards quite limpid, flowed out. The tension of the swelling was considerably diminished. Next day, after a quiet good night, the swelling was found to be again as prominent as ever, and it was observed that the fontanelles were unusually depressed. A second puncture was made, and the tumor became now so sunk and flaccid, that the edges of the fissure in the vertebræ could be distinctly felt. On the following day, the operation was performed twice! The infant became restless, uneasy, and pale; slight convulsions came on, and it died on the fourth day after the first puncture had been made.

On dissection, a quantity of limpid serosity was found in the sac, and in the ventricles of the brain. The spinous processes of the last lumbar and of the first sacral vertebræ were awanting; there was no spinal canal, nor yet any spinal foramina in the sacrum, and the cauda equina hung out of the sac, and lost itself in its thick loose and white walls.—*Hannoversche Annalen.*

*Remarks.*—Our object, in reporting the preceding case, is chiefly for the purpose of directing our readers' attention to the nature of the congenital malformation known by the name of Spina bifida, and to the proper treatment of it. There is nothing unusual or extraordinary in the case we have extracted from the German periodical; nor would it have merited notice, except in so far as it affords us an opportunity of pointing out the extreme impropriety of the treatment which was pursued.

The medical man appears to have acted, as if he had a simple encysted watery tumor to deal with; and he therefore very probably imagined that the mere evacuation of the serosity could not possibly do any harm. Very different is the nature of Spina bifida. It is one of the most serious congenital anomalies or malformations, which can happen to a child. There is an actual defect of the spinous processes and posterior part of the spinal arch of one or of more vertebræ. The spinal marrow is thus exposed, and generally protrudes through the fissure, in consequence of its sheath being distended with serosity. The mere discharge of this serosity may diminish indeed the size of the swelling; but this is, as it were, the mere outwork of the disease. The 'fons et origo' of the mischief is the defect in the formation of the vertebræ.

These bones have been—in consequence of some cause or another, the nature of which we do not at all understand—arrested in their progress to mature development, and have not closed or coalesced in their posterior part, thus leaving the spinal cord exposed. We have already alluded to this subject in the article on philosophical anatomy in the present number of this Review, and need not again dwell on the very curious and most interesting topic, as illustrative of the centripetal development of the vertebræ, of their primitive duality, and of their subsequent coalescence in the median line. At an early period of embryotic life there was, so to speak, a spina bifida along the whole course of the spine; all the vertebræ were open behind, and the spinal cord was exposed from the cranium to the sacrum. The formation of the spinous processes is the latest step in the development of the vertebræ. Now if from some cause this development is stopped or impeded at any part of the column, while the rest of the column meets with no stoppage or impediment, then the child at birth exhibits a Spina bifida at that part.

This is the simple and rational explanation of this congenital defect. What adds very much to the seriousness of the malformation—a consideration which ought necessarily to influence our prognosis—is the circumstance of almost all infants born with spina bifida being found to have some other defect or anomaly. Thus it has been observed that hare-lip is very frequently co-existent with this affection. A tendency to hydrocephalus chronicus, indicated by a large head, open state of the sutures is also a very common complication. It is most important therefore to bear in mind that a congenital malformation like spina bifida ought to be viewed, not simply as a mere local anomaly, but rather as one proof, among others, of a tendency in the constitution to imperfect development.

The vital powers of a child affected with spina bifida, chronic hydrocephalus, or even with the less serious congenital malformations of hare-lip, fissured palate, umbilical hernia and such like (all of these being examples of imperfect coalescence of parts, which are normally separated from each other at an early period of fœtal existence), are very generally more feeble, and much more apt to give way under disease, than those of perfectly-formed infants. Very many of them sink long before the process of dentition commences; and those, who do survive until this period, almost universally suffer long and most severely. This hint ought not to be lost on the practical physician. Let him examine attentively those children who are very backward in cutting their teeth, and who are more subject than others to fits, &c. at this time, and he will find that they are generally pale and feeble children, whose heads are large, or at least in whom the fontanelles are more open than usual, who are affected with umbilical hernia, in whom the testicles have not descended, or who, in short, exhibit some mark or another of retarded development and imperfect growth.

Such being the case, it is almost unnecessary to state that the treatment of all such congenital defects, as spina bifida, requires the greatest caution and delicacy. It is not by merely discharging the fluid of the tumor, that we can ever rationally hope to cure this malformation. In most cases, we believe that the best-directed efforts of the scientific physician will prove abortive, and that the infant will not survive many months. They generally go off in a paroxysm of convulsions. Certainly in no case is the treatment, which was so injudiciously followed in the preceding case, to be adopted. In the course of *four* days, to perform *four* operations!! This is really mail-coach speed in surgery. Even had the swelling been a simple abscess, or encysted watery tumor, such rapidity of execution would have been most reprehensible. But in so serious an affection as spina bifida it was positively murderous.

When we determine to draw off the serum, the puncture should be made with a fine needle at several points, rather than with a larger instrument at one point. It is much safer that the fluid should ooze slowly out, than be discharged quickly. Gentle and uniform pressure should be kept up for a length of time; and one, or even several weeks, according to circumstances, ought to elapse, before we have recourse to another operation. Meanwhile the greatest attention is to be paid to the health of the infant, and all judicious means resorted to, for the purpose of invigorating the constitution, and assisting nature to carry on the reparative process.—*Rev.*

---

IMPERFORATE ANUS. SUCCESSFUL FORMATION OF AN ARTIFICIAL ANUS IN THE RIGHT GROIN.

An infant, three days and a half old, was found to have no anal aperture. The raphé of the perineum extended from the scrotum to the point of the os coccygis, without any interruption.—The child was jaundiced all over; the abdomen was hard and distended; there had been no vomiting, and of course no alvine evacuation. It had taken the breast repeatedly, and had also voided urine several times. An incision was made at the usual place of the anus, and a trocar was introduced for at least an inch or more in the direction of the rectum; but without any effect. It was, therefore, deemed unsafe to penetrate to a greater depth; and a proposal was made to open the cæcum in the right iliac region. This was accordingly done. An incision was made through the integuments immediately in front of the anterior superior spinous process of the os ilii, and when the peritoneum was opened, a portion of small intestine escaped. This was replaced, and the caput cæci being found, an incision was made into it. Several ounces of a consistent meconial matter immediately flowed out, with great relief to the symptoms. The progress of the case was most satisfactory (der Fortgang war ganz erwünscht); copious alvine evacuations began and continued to escape. On the eighth day after the operation, the stitches of the abdominal wound were removed.

In a second case of congenital obstruction of the bowels, in which however the anus was quite normal, and the rectum was pervious as far as the finger or catheter could reach, the operation of establishing an artificial anus in the right groin was not acceded to by the parents. The infant died on the third day after birth; and, on dissection, the whole extent of the large intestine was found to be so much contracted, that it did not exceed in size that of a large goose-quill. When slit open, it was filled up with a membranous-looking stuff of a yellowish white colour, which adhered very firmly to the walls of the gut. The valvula coli was much contracted. The inferior extremity of the ilium was distended with thick meconium; the rest of the small intestines, and also the stomach, contained an offensive fluid of a yellowish-green colour.—*Medizin. Zeitung für Heilk: in Preussen.*

---

CASES ILLUSTRATIVE OF PUERPERAL FEVER.

CASE 1.—*Abortion—Phlebitis of the Hypogastric and Iliac Veins and of the Vena Cava—Purulent Arthritis.—Dissection.*

A washerwoman, 44 years of age, was admitted into the Maternité on the 8th of January, 1835, three weeks after she had miscarried. She mentioned that she went out to her work on the fourth day, and caught cold; that she had then suffered from flying pains through the abdomen, and that the lochia had entirely ceased from that date. A few days subsequently she experienced a smart rheumatic attack in the left knee and elbow, attended with general feverishness. The affected joints became more painful, in spite of free leeching. When admitted into the Hospice, she complained much of the left elbow joint, the slightest movement of which occasioned severe agony. The left knee also was very painful, and was much swollen, apparently from an effusion into the synovial capsule; the patella being raised up, and the distended capsule swelling out on each side of it. The skin covering the joint was red and inflamed in patches. The abdomen was free from uneasiness, even on pressure, although the bowels were relaxed. The pulse was 116; the respiration was frequent, but not embarrassed; the skin was hot; the tongue was parched and red at the tip and corners; and there was a continual wakefulness, but no tendency to delirium. The patient was bled to 14 oz. — laudanised cataplasms were applied to the affected joints, and a grain of opium was administered. On the following day the blood drawn was found to be strongly buffy; the left shoulder-joint had become painful and inflamed, and the diarrhœa had increased.

It is unnecessary to detail minutely the progress of the case. The prostration of all the vital powers increased daily; the abdomen became tympanitic, the tongue was covered with a dry brown crust; the urine and fæces were sometimes passed involuntarily, and the patient was frequently delirious. On the 22d (the fourteenth after admission), she was seized with a violent fit of shivering, which lasted for many hours, and was followed by profuse sweating. She lay almost insensible, being generally either delirious or comatose.

She lingered on for eight days more; the affected joints having remained in the same painful swollen state to the last. Mercurial frictions on the abdomen constituted the chief part of the treatment after the 16th day of the month, when M. Rostan, under whose care the patient was, began to suspect that her disease was not ordinary rheumatism, but rather that singular and very fatal form of arthritis, so often connected with phlebitis, and in the present case with that form of it which is apt to follow metro-peritonitis.

*Dissection.* The affected joints were first examined. The cellular substance around the left shoulder-joint was infiltrated with pus; but none was found within the articular capsule. The capsule was not discoloured, but contained within a synovial fluid of a more yellow hue, and of a more viscid consistence than in health.

The elbow-joint contained a small quantity of well-formed pus: the surface of the capsule exhibited a granular appearance, and here and there a patch of "rougeur pointillée." A larger quantity of purulent matter was found in the knee-joint, and its synovial membrane was observed to be in part raised or detached on the inter-articular cartilages, and elsewhere to be red in patches. There was no trace of pus in the surrounding cellular substance; and the muscles of the thigh and leg were quite healthy.

The cephalic and thoracic viscera did not present any decidedly morbid appearances. In the lungs, although they were congested towards their inferior and posterior parts, there were none of the purulent deposits, nor any of those circumscribed engorgements which have been so well described by M. Dance, and which precede the formation of metastatic abscesses in the pulmonary tissue.

The liver was equally free from such lesions. However, on the upper surface of the spleen there was a small abscess filled with well-formed pus: the tissue of the viscus itself was not sensibly altered, except at one point, about the middle of its anterior surface. There, it was of a brown colour, of a firmer consistence than elsewhere, and it was easy to perceive that numerous vessels converged towards this point. We may, therefore, regard the change as the result of a circumscribed engorgement.

The uterus—its muscular tissue and its blood-vessels—was minutely examined; but no decided traces of disease were discovered. The veins however which traversed the broad ligaments, especially those at the lower parts, were greatly indurated, so that they gaped like arteries, when divided. The cavities of these, and also of the hypogastric veins, were filled with yellowish grey fibrinous clots, which adhered very strongly to the parietes. These changes, the induration of the parietes, and the partial obstruction with clots—were traced along the hypogastric and common iliac veins to the vena cava, which was found to be much larger than usual. Its parietes were greatly thickened from the point of bifurcation to where it passes behind the liver; and, on slitting it open, its cavity was found occupied partly with thick pseudo-membranous concretions, which adhered strongly to the parietes, giving them a granulated aspect, and partly by a long coagulum, in the centre of which was purulent matter. Some of the veins also of the ovaries were similarly diseased as those which we have described.

*Remarks.*—The case, which we have now detailed, is one of great interest in a practical, as well as in a pathological point of view.

The disease in its earlier stages appeared to be ordinary rheumatism; but the great prostration—not an ordinary symptom of this disease—the gradual aggravation of all the constitutional distress, and the obstinacy of the local sufferings, excited the suspicions of M. Rostan that there was a deep-seated mischief going on somewhere. The history of the case, the previous miscarriage, and the invasion of the shivering fits, led him to pronounce it to be chronic metro-peritonitis.

The pathologist will not fail to notice the very limited extent of visceral lesion in the present case, considering the number and the importance of the veins so seriously diseased. Dance, in his able memoir, has alluded to this discordance between the extent of the phlebitis, and the number of the secondary suppurations. In some of the cases recorded by him, as well as by other pathologists, there were found abscesses not only in several of the joints, but also in many of the muscles and viscera, when only a few inches of one or two inconsiderable veins were inflamed; and here, in the present case, the vena cava itself and some of its most important branches were deeply diseased, and yet the only viscus, which exhibited any traces of secondary suppuration, was the spleen.

Another topic for consideration, suggested by this dissection is, where did the phlebitis commence? The veins of the substance of the uterus did not present any traces of morbid change. These appeared first in the veins of the broad ligaments, and extended from them into the hypogastric veins. Are we to suppose that the phlebitis commenced in the veins of the broad ligaments without previously affecting those of the uterus itself?

M. Dance was of opinion that in all such cases the inflammation '*debutait*' in the uterine sinuses, and was propagated along these to the remoter branches. We must confess that we hesitate quite to assent to this doctrine, in the very face of such dissections as the present; and the chief reason for our hesitation is, that 'quand des veines ont été enflammées au point de donner lieu à suppuration de leur parois, ces veines s'obliterent, et c'est par ce seul mecanisme que s'opere la guerison.' This obliteration had taken place in the following two cases, which also occurred in the Maternité Hospice.

Case 2. — *Metro-peritonitis, which disappeared—chronic enteritis—death—ulcerations of the intestines and uterine phlebitis.*

This patient, a girl 19 years of age, was seized with pain in the hypogastric region, towards the iliac fossæ, on the third day after accouchement. Feverish symptoms also made their appearance; but the repeated application of a number of leeches induced a decided relief. The bowels however became relaxed, and along with a return of the abdominal tenderness, which was now diffused over the whole belly, an unexpected prostration of strength was remarked. The pulse had become frequent, small and feeble, and the patient was much distressed with the repeated evacuations from the bowels. Mercurial ointment was ordered to be rubbed freely over the whole abdomen, and opiate enemata were administered. The patient lingered for nearly five weeks. The diarrhœa, although mitigated, had never been quite checked, and an almost constant state of feverishness had existed during the whole time.

*Dissection.*—The lungs were sound, with the exception of having a few tubercular depositions. The parenchymatous viscera of the abdomen were free from disease. The large intestines were throughout much thickened, and in various points ulcerated. On examining the uterus, parts of its substance were found to be healthy, and other parts to be, as it were, ecchymosed.

On its anterior surface, near to the insertion of the broad ligaments, there were several firm, cylindrical, and tortuous cords, which, when divided, proved to be veins plugged up with 'pus concret solide, analogue à une fausse membrane, dont l'organization est dejà fort avancèe.' The right ovary appeared to be quite atrophied, and the left one was much smaller than usual. (Here the report of the dissection most abruptly ceases. Nothing is said of the state of the larger veins.)—*Rev.*

Case 3.—*Metro-peritonitis followed by chronic enteritis — ulcerations in the large intestines, and traces of cured phlebitis.*

A young girl was safely delivered of her first child after an easy labour. For the following two days she was going on well. On the third she was suddenly taken with a shivering fit, which was followed by pain and tension in the hypogastrium.

On the next day the features were much altered, the decubitus (position in bed) was dorsal, the abdomen was much distended, *apparently from effusion having taken place.* The bowels were purged, the lochia were scanty, and there was no secretion of milk. Prescription.—Riz avec sirop de gomme, trois pots. Potion gommeuse avec diascordium. Deux vesicatoires aux cuissés. Ongment mercuriel ℥iij. pour douze frictions. Bain de siege. Demi-lavement d'amidon, et de pavot avec laudanum de Rousseau, gout iv.

The progress of this case was much more rapid than that of the preceding one, for the poor girl died after a week's suffering.

*Dissection.*—The cephalic and thoracic contents healthy. *No effusion within the abdomen.* The small intestines but very slightly altered; the colon thickened, and its mucous surface inflamed in patches and exhibiting a thin layer of fibrinous exsudation.

When this layer was removed with the edge of the scalpel, the mucous membrane was found ulcerated. The blood-vessels at the sides of the uterus and within the folds of the broad ligaments were impervious, from being filled up with 'pus concreté analogue aux fausses membranes,' which firmly adhered to their parietes. These obstructed vessels could be traced through the substance of the uterus, but not beyond the broad ligaments, and the larger venous trunks were free from any change.

In another case, where the patient had died three weeks after her accouchement which had been followed by violent metro-peritonitis, the following remarkable appearances were found on dissection. There were gangrenous eschars over the trochanters and sacrum. The cellular substance around these bones was infiltrated with purulent matter, and the extremity of the sacrum and the coccyx were denuded. The sacro-coccygean joint was filled with pus and the ligaments were destroyed.

The cephalic, thoracic, and chylopoietic viscera were sound. The uterus was glued by fibrinous filaments, anteriorly to the omentum and posteriorly to the rectum.

On the left side, the round ligament, the ovary, and the Fallopian tube were bound down by strong adhesions. When the substance of the uterus was cut into, numerous yellowish-white points were seen. These could be easily traced in different directions, and proved to be caused by the uterine veins filled with 'un pus pseudo-membraneux.' This lesion of the veins did not extend beyond the broad ligaments.

From this, and from the preceding two cases we are taught the process, by which the cure of uterine phlebitis is effected. The affected vessels become plugged up with coagulable lymph, and are converted into impervious cylinders, so that the pus cannot be transmitted into the larger trunks.—*Archives Generales.*

### LAMENTABLE CASE OF DIFFICULT PARTURITION. DISGRACEFUL CONDUCT OF M. ROUX, AT THE HÔTEL DIEU.

We can scarcely trust ourselves to express our opinion of the inhuman conduct of M. Roux, one of the leading surgeons in Paris, and successor of Dupuytren at the largest hospital there. The mere facts of the case will abundantly speak for themselves. A woman, 28 years of age, was admitted into the Hôtel Dieu at three o'clock in the afternoon, having been taken with labour-pains on the preceding evening at seven o'clock. She was of very short stature, (scarcely four feet) and of a "constitution eminemment rachitique." She had been pregnant twice before. Her first child was delivered by the forceps, after a labour of twenty hours. Her second had at the full period been born without much difficulty, and by the unassisted efforts of the uterus. The midwife, who attended her in her last labour, having this time waited in vain for the advancement of the child's head, called in the assistance of several physicians. Three came, and the forceps was applied "sans resultat." She was then taken to the Hôtel Dieu. The membranes had been ruptured in the attempts to apply the forceps, the labia were swollen and painful, the vagina was tender, and the cervix uteri was lacerated. The head of the child had scarcely entered the cavity of the pelvis, and, just below and somewhat behind it, the promontory of the sacrum was felt to be unusually projecting. The reporter of the case says that, in order to ascertain the exact position and state of all the parts, he introduced his entire hand, and from this examination he inferred that the conjugate diameter of the inlet was about two inches and a quarter. When M. Roux examined, he was at first doubtful whether it was the head which presented—it felt to be so much elongated and flattened. Under these circumstances (!!) he resolved to apply the forceps. The introduction of the blades was most difficult, tedious and painful. He made several attempts to extract, but all in vain. At length "pressé par le temps! obligé de se rendre au concours de l'ecole où il est juge!! et desesperant d'en venir au bout!!!" he left his wretched patient with the forceps in her body, promising to send to her assistance his son-in-law, M. Danyon, "who," said he, "understands better than I do these sorts of operations." M. Danyon however was not to be found, and it was not till nearly two hours afterwards that M. Roux returned, accompanied by M. Dubois. He (M. Dubois) immediately removed the forceps, and extracted the child by embryotomy. The poor woman died in 30 hours afterwards.

Such is the reported history of this awful case, and in confirmation of its truth we are informed that M. Dubois, in his clinical lectures, "a raconté lui-meme ce fait et en a confirmé toutes les particularités."—*La Lançette Française.*

We shall offer no comment on the above description. We much fear that such cases are not uncommon in France, as well as in other parts of the Continent; for, despite the high names of Dubois, Capuron, Siebold, &c., the state of midwifery is truly lamentable, in even the neighbourhoods of Paris and Vienna.

The treatment of the foregoing case was simple and most obvious. The patient ought to have been bled freely, and the os uteri allowed to become freely dilated. If, when the head of the child had fairly entered the brim of the pelvis, the forceps could be easily applied, all very well;—but no force ought to have been used; and if these attempts had failed, and speedy delivery had been deemed requisite, recourse should at once have been had to embryotomy. Our readers know well the high opinion which we have of Dr. Collins' treatise, reviewed in our number for last July. The precepts there inculcated, "Nocturnâ versate manu, versate diurnâ.—*Rev.*

---

### Professor Naegele on Difficult Parturition from the Agglutination of the Os Uteri.

Among the occasional impediments to an easy delivery, most obstetric writers have enumerated the occlusion of the os uteri, in consequence of previous inflammation, or of callosities, scirrhous indurations, cicatrices, &c.

This occlusion may be either complete, or only partial. In some cases it is so complete, that all traces of the orifice are almost obliterated, and great difficulty is experienced in ascertaining exactly its position. But this is an extreme case, and few examples of it are recorded by any author. The lesser degrees of this lesion are not unfrequent; and perhaps they are more common than is generally supposed, as it is not improbable that a certain degree of morbid agglutination of the os uteri may exist during the early period of labour, and may gradually give way to the contractions of the womb during labour, before the accoucheur has made any examination.

Madame La Chapelle has alluded to some varieties of the occlusion of the os uteri in her valuable treatise on midwifery.

" We have sometimes," says this famous sage-femme, "found it difficult to discover the external or vaginal orifice of the uterus, in consequence of its being closed up by thick gelatinous mucosities. The edges of the orifice may be so strongly agglutinated, that it resists for a great length of time the parturient efforts of the uterus. When this is the case, it is not unfrequent that the parietes of the uterus near its cervix become so much extenuated, and the head of the child is thereby felt so distinctly, that the accoucheur may suppose that the membranes only intervene between it and his finger, while the os uteri is not at all open." We have reason to suspect the occurrence of this accident, when, at the commencement of labour, the inferior segment of the uterus descends very deeply into the pelvic cavity, and yet its orifice cannot be easily discovered; or when the orifice feels to the finger like a fold, or cup-like hollow, somewhat depressed in the centre, and not corresponding to the axis of the pelvis. In proportion as the uterine contractions during labour acquire more force, the lower segment of the uterus is pushed more and more down into the pelvic cavity, and its parietes become excessively stretched and thin, so that they resemble to the touch rather the mere membranous envelopes of the fœtus, than the muscular walls of such an organ as the uterus.

This extreme degree of attenuation of the uterine parietes may lead the inexperienced accoucheur to commit the awkward and very dangerous mistake of endeavouring to rupture them in a tedious labour, supposing them to be the protruding membranes.

The only safe rule to follow, is to avoid all manual interference, unless the os uteri can be distinctly and indubitably ascertained.

Notwithstanding the severity and the continuance of the pains, the uterine orifice may not only remain firmly closed, but may also become more and more elevated, and be carried at the same time more to one side of the pelvis than the other. After the pains have existed for a length of time, and when the energies and strength of the poor sufferer appear almost quite exhausted, Nature will sometimes induce a gradual relaxation and opening of the orifice. If this favorable event does not take place by the mere efforts of Nature, or with the assistance of art, the uterus will either rupture, or become totally paralysed and powerless.

The cause of the agglutination, to which we are now alluding, is in most cases a previous inflammation of the cervix uteri. Often when there is occlusion of the orifice, there is also a coexistent prolapsus uteri, and generally more or less of leucorrhœa. The inflammation may have proceeded so far as to have induced an ulcerated state of the cervix, accompanied with an exsudation of coagulable lymph round the edges, causing them to adhere. These serious changes sometimes take place, without having excited any very obvious symptoms at the period of their formation.

The pseudo-membranous and fibrous tissue, which constitutes the bond of agglutination, is quite similar to "cette matiere qui sert de moyen d'union entre la placenta et la matrice, les poumons avec la pleure costale, et les intestines entr'eux, on avec la paroi abdominale lorsque l'inflammation se termine par adherence."

It is not easy to determine at what period of gestation the agglutination, unconnected with any lesion of structure of the cervix or of the os uteri, generally commences. In one case which occurred in the practice of Naegele, the orifice was quite patent six weeks before delivery. Bartholin seems to have observed the agglutination, of which we are speaking, in the early months of pregnancy. A woman, five months enceinte, was affected with paraplegia from the haunches downwards. As there was no chance of saving the life of the mother, an attempt was made to extract the child by force; but in spite of every effort, the accoucheur "could not penetrate into the uterus." On dissection the uterine orifice was found firmly plugged up. [Is there any thing extraordinary in this?]—*Rev.*

The agglutination of the uterine orifice is to be distinguished from the "occlusion de ce canal," when the cervix has contracted adhesions with the posterior wall of the vagina. In the latter case, the orifice is not to be felt at that part of the uterus which is protruded into the hollow of the pelvis. The traces of an inflammation, of ulcerations, of cicatrices, indurations, &c., and the escaping of the waters—which cannot take place when the cervix uteri adheres to the vagina—will serve to render the diagnosis more satisfactory. The agglutination of the cervix cannot be confounded with any contraction of this part induced by cicatrices, or by scirrhosities; for the finger, or, if necessary, the eye will always, in the latter cases, detect, either at the orifice or in the adjacent structures, the true nature of these changes.

In all such examinations, the accoucheur should never permit himself to be satisfied, unless he can feel distinctly, and unequivocally ascertain the os uteri. We have known more than one instance in which the uterine parietes, stretched and attenuated by the inclosed liquor amnii and child, have been mistaken for the mere enveloping fœtal membranes. Much injury may be inflicted by a rash practitioner who has made this mistake. With respect to the prognosis in cases of the agglutination of the uterine orifice, we may very generally consider it to be favourable, provided the accident has been ascertained sufficiently early, and the treatment pursued has been judicious. Perhaps the chief source of the danger, is the possible exhaustion of the woman's energies, during the early stage of labour before the mouth of the uterus begins to dilate.

If the obstruction be very unyielding, and be not relieved in time, palsy and even rupture of the uterus may ensue. But such events will never happen in the practice of a well-informed accoucheur. If he has reason to suppose that agglutination of the os uteri is the cause of the protracted and inefficient labour-pains, he will introduce his fore-finger, or a bougie, or female catheter into the orifice, and with gentle pressure rupture the opposing adventitious membrane. A few drops of blood usually follow this operation. The finger is to be preferred in most instances to any instrument. There, is however, very little risk of rupturing the fœtal envelopes even with the bougie.

Some accoucheurs have most improperly recommended that the obstruction be overcome by incision; but such practice is not only quite unnecessary, but is always dangerous.

In confirmation of the preceding statements and opinions, Professor Naegele has adduced the following cases.

A woman, 38 years of age, pregnant with her third child, was admitted into the Heidelberg Midwifery Hospital.—On examination it was found that there was a prolapsus of the right wall of the vagina. Labour commenced on the evening of the 10th November. The liquor amnii had been draining off since the preceding day, before the pains had commenced. The uterine contractions had continued during all the night and the following day, and although they were strong and even violent, the os uteri remained obstinately closed. The orifice was directed backwards, and somewhat to the left side, and presented no trace of distinct labia, but only a transverse slit, or aperture with a smooth and unresisting margin. During the second night the labour-pains ceased altogether, and the patient slept. On the 12th no progress was made. When the cervix uteri was depressed with the finger, it was found that the os uteri had not become at all dilated in consequence of its extreme rigidity, and that by pushing the finger on with gentle force, "une trame filamenteuse," which had held the edges of the uterine orifice agglutinated, gave way under the pressure. From this moment, the orifice, although the pains were very feeble, began to expand: they continued all the afternoon and following night, and on the following morning, the woman was safely delivered of a dead child.

Portal, in his Pratique des Accouchemens, published in 1685, has related a very similar case. The labour having been unusually tedious, he found on examination that by pressing the finger on the os uteri, which at the time was not at all dilated, a membrane as tough as parchment gradually gave way. The pains thereupon speedily became effective, and delivery was safely completed.

Sandifort in his Thesaurus Dissertationum has given the particulars of the post-mortem appearances found in a case which had occurred in his practice. "The cervix uteri was situated opposite to the second sacral vertebra, and felt much attenuated. On the side of the uterine cavity it readily admitted the point of the fore-finger; but on that of the vagina, it was so firmly closed that even a common quill could not be introduced, and the resistance could not be overcome by even 'une forte impulsion.' It seemed to be of an almost aponeurotic character."

In the Transactions of the Swedish Academy, the following case is recorded. A woman had been for eight days in labour. The pains continued to be violent in spite of four bleedings, which had been practised. The lower segment of the uterus had become so much stretched and depressed, that it was at first taken for the protruding bag of the membranes. In the centre of it however a small slit, plugged up by fleshy filaments, was to be felt. By introducing the point of a female catheter into this slit, these filaments were readily ruptured. A few drops of blood followed. The labour-pains continuing, the os uteri began soon to dilate, and delivery was completed within a short time.

The narrator of this case attributed the agglutination to an inflammatory affection of the vagina and neck of the womb, which had occurred about three weeks before labour.

In another case related in the same work, the symptoms were very similar. After the labour-pains had continued for two days, an examination was made. The lower part of the uterus was excessively distended and attenuated, and in spite of the most accurate examination, no distinct trace of the os uteri was discoverable. At the lower and posterior part a small fold, not bigger than a barley-corn, was to be felt; and this was presumed to be the uterine orifice. A female catheter was conveyed along the finger to this point and pressed firmly against it, so as to rupture the 'fibres charnues,' which closed up the opening. The finger was then gradually passed through it; and, soon afterwards, strong labour-pains came on and effected the delivery of the child.

In the second vol. of Siebold's Journal für Geburtshelfe an instructive case of agglutination of the os uteri is given. The woman had been 48 hours in labour, when the physician was called. Neither he, nor another practitioner could detect the os uteri, and the head of the fœtus was felt so distinctly that, at first they mistook the attenuated uterine parietes for the protruded bag of membranes.

On the following day, another examination having been instituted, a small opening, obstructed by a membrane which gave way upon pressure with the point of the finger, was to be indistinctly felt. Behind this membrane, the os uteri was found dilated to the width of more than an inch.

In the Med. Chirurg. Zeitung for 1821, an instance of agglutination is communicated by Dr. Rainer, who employed a bistoury to divide the contracted orifice, and was afterwards obliged to have recourse to the forceps to extract the child. The patient recovered speedily and well. (Surely the German ladies can stand rougher usage, under the hands of their accoucheurs, than those of other countries.) —*Rev.*

Two other cases, where recourse was had to the use of a cutting instrument to divide the obstructing membrane and part of the cervix uteri, are mentioned by Naegele in his memoir; but, as he himself condemns the practice, we need not communicate the particulars, except only to state that both of the patients recovered without any unpleasant symptoms.—*Mogostocia e conglutinatione orificii uteri externi.—Auct. H. F. Naegele*, 1835.

The preceding remarks from the pen of so distinguished an obstetrical writer as Professor Naegele may be deemed worthy of the attention of those, who engage in midwifery pursuits. We must however confess that, in our own practice, we have seldom met with cases of dystocia or difficult labour, where the impediment might be properly attributed to a mere adhesion or agglutination of the uterine orifice in consequence of a previous exsudation of lymph, and of the formation of a connecting adventitious membrane; but we are unwilling to oppose our own opinion to that of the celebrated German. Certain it is that, we do meet occasionally with cases, in which the uterine tumor becomes much depressed in the pelvis, and the walls of the uterus become so very thin, that they are apt to be mistaken for the protruding bag of the membranes, the os uteri remaining, all the while, firmly closed. The inexperienced accoucheur requires to be put on his guard against this accident; and his first and foremost duty therefore on all occasions, is to ascertain the position and state of the orifice.—*Rev.*

---

TREATMENT OF THE INSANE.

The pamphlet, from which we extract the following observations, is one of those lately sent to us by Dr. Warburton of Palermo. The regulations, medical and economical, of the Royal Lunatic Asylum instituted there about ten years ago, are truly excellent. They were drawn up by Dr. Pisani, the director and chief physician of the establishment. We are pleased to find that the use of fetters and of all harsh restraint has been entirely abolished, and that the mildest means of confinement only are ever resorted to. The sixth article runs thus:—"Although it is a principle now universally recognised, that the method of treating and curing insanity ought to be founded chiefly on moral management and discipline of the patient, we are not to forget the important aids which may be derived from judicious medication. It is therefore quite indispensable that in a lunatic establishment there should be resident physicians, who are intimately acquainted with the physique of man, and who know how to adopt, in each particular case, all those indirect curative means, such as local and general bleeding, the use of blisters, of the douche bath, &c. which skill and science may suggest. It is of great importance that, the insane themselves should be convinced that the cure and management of their cases are intrusted to competent medical men, as the idea, that insanity is an incurable disease, has been far too generally prevalent among the vulgar."

In a subsequent article we find the following excellent observations: "The moral cure or management is the chief, perhaps the only, method of curing the insane. The basis of this method is seclusion or separation, which has been found to act in a direct and powerful manner on the operations of the mind. When a patient is admitted into the institution, all intercourse with his relatives is interdicted. The presence of them almost invariably excites violent commotion, and renders the characters of the insane much more intractable. A long course of observation has convinced us that the insane never recover their reason completely, in the bosom of their families. A change of scene and objects will generally produce in time a change of ideas."

There is a laudable humanity and kindness in the regulation which forbids the employment of the words "insane," "mad," (pazzo, folle, matto,) in the institution, and which has substituted the title of superintendent for that of master or keeper. This hint may be very becomingly adopted in our own country. How often do we hear of the unfortunate inmates of asylums reproaching their friends for their unkindness, and protesting that they are not *mad*. It is an offensive epithet, and often, we are certain, aggravates the excitement of the patient.

Dr. Pisani recommends in strong terms that the insane should be encouraged to take regular and daily exercise, as walking, gardening, and playing at ball and racket, &c. in the open air, and to occupy himself with innocent recreations and amusement within doors, more especially music, cards, billiards, &c. The female patients are encouraged to assist in the labours of the kitchen, dairy, and washing-house.—Dr. P. suggests the propriety of always rewarding the invalids with some small remuneration for every task, which they may do.

The resident medical officer has four pupils attached to his service, and it is the duty of these to draw up the reports of each case on admission, and to perform all the minor operations which may be deemed necessary. The bodies of those who die in the institution are all carefully dissected, and the crania, and any other part, which is thought worthy of especial notice, are preserved in the museum.

A chaplain is attached to and resides in the institution. His duty is to celebrate the mass every day to the insane, to assist them in all their religious observances, and to administer extreme unction to the dying.

We have already stated that all harsh means of restraint or punishment have been abolished by the directions of Dr. Pisani. The only modes of confinement which he ever employs is the use of the straight-jacket, and seclusion in a solitary room. The rotatory chair, the surprise-baths, &c. are prohibited. The use of a continued stream of cold water on the head, along with the restraint of a straight-jacket, will be found sufficient to tame the most furious maniacs.

Our limits prevent us from making any more extracts from these "instruzioni;" but we feel the sincerest pleasure in recommending them, as forming an admirable code of rules and regulations for the management of a lunatic establishment.

---

### Professor Lallemand on Spermatorrhea.

The distinguished Professor of Montpelier has recently published a treatise on involuntary seminal emissions, in which he has minutely described the symptoms, most frequent causes, the pathology, and the treatment of this most insidious and offensive disease. He states:—

"In the course of the last 14 years I have collected upwards of 1500 cases of involuntary spermatorrhea, in all of which the health was seriously affected, and in many death was the consequence. The greater number of the patients had been directed to me, *for supposed cerebral affections*. In not a few of the cases, it had been supposed that the patient suffered from chronic gastritis, or gastro-enteritis, from aneurism of the heart, from incipient phthisis, or from some of the multifarious forms of nervous disturbance, and most frequently from hypochondriasis."

The cases, adduced by M. Lallemand, exhibit examples of persons, whose constitutions had been vigorous and robust, presenting at the period of life when the body has acquired its development, and without any appreciable cause, all the symptoms of physical exhaustion, associated with a very marked feebleness of the mental powers.

In examining attentively the probable cause of this premature decrepitude, it was frequently discovered in an existing spermatorrhea, or seminal weakness. The opinion of the author, who recognises in a protracted spermatorrhea the cause of numerous encephalic distresses, is confirmed by the consideration that, in some fatal cases where cerebral congestion, the decay or perversion of the intellectual faculties, the exhaustion of the physical energies, &c. have existed for a length of time, no signs of morbid change, except in the genito-urinary organs, are discoverable on dissection. The most frequent and the most potent cause of spermatorrhœa is, according to Lallemand, an inflammation affecting the secretory and excretory apparatus of the semen—an inflammation which, commencing in the urethra and prostate gland, is apt to extend to the vasa efferentia, vesiculæ seminales, vasa deferentia and testicle, as well as to the urinary bladder.

---

### On the Curative Properties of Chlorine, and of the Chloride of Lime.

In *Typhus Abdominalis*. Dr. Truse has for many years derived great advantage in his practice from using the aqua oxymuriatica, or liquid chlorine, in almost all cases of typhus fever.

He exhibits a drachm or more of it every two hours, in combination with syrup and some mucilaginous decoction. In addition to the use of this means, he applies leeches to the epigastrium, sponges the surface of the body and limbs with cold vinegar and water, directs cold lotions to the head, and rubs the abdomen with mercurial ointment. The use of the aqua oxymuriatica—an ounce or so in 24 hours—may be continued for eight or ten days; and then exchanged for some light bitter tonic.

In *Intermittent fever*. The form of this pyrexia, in which Dr. T. has found most benefit from the use of the chlorine, is the irregular or illegitimate variety of ague, in which the paroxysms do not return regularly, and the intermissions are not entirely free from feverishness. Under these circumstances the bark will often fail; and for this reason, that the disease is not genuine *intermittent*, but rather disguised *remittent* fever. After the chlorine has been continued for a few days, it generally happens that a distinct and well-marked paroxysm of fever takes place, and this is succeeded by a complete pyrexia.

In *Gastric Fevers*. The aqua oxymuriatica is so admirable a remedy in all cases of this fever, that often there is no occasion to have recourse to any other medicine for its speedy cure. When the fever has reached its second stage, the chlorine may be most advantageously combined with small doses of rhubarb and ginger, as in the following formula.

℞. Rad. rhei, ʒj. Rad. zingiberis, ʒss. infunde in unciis sex aquæ ferventis. Colat. admisce aq. oxymuriat. ℥j. Oleisacchari menth. piperit. ℥ss. Misce. A table spoonful may be given for a dose frequently.

In *the Exanthemata*. The oxymuriatic acid appears to possess, in addition to its admirable effects in allaying the febrile exacerbations, a remarkable power against the principle of the contagion (ansteckungs-stoff.) Our author praises its efficacy more especially in scarlet-fever. In variola he recommends not only its internal, but also its external use. It may be applied either as a wash, or as a liniment with oil.

The chloride of lime is a most serviceable application in a great variety of outward sores. It forms an excellent gargle for foul ulcers of the mouth. For this purpose, eight ounces of the decoction of rhatany root may be poured on two drachms of the powdered chloride.

In almost all torpid, phagedenic, scrofulous and herpetic sores, the solution—three or four drachms dissolved in a pound of water—will be found to be a most convenient and useful application.

In varicose ulcers the best treatment consists in applying the chloride solution during the day, and in sprinkling the surface of the sore with lapis calaminaris during the night.

